# Systematic Review and Bayesian Meta-analysis of the Dose-response Relationship between Folic Acid Intake and Changes in Blood Folate Concentrations

**DOI:** 10.3390/nu11010071

**Published:** 2019-01-02

**Authors:** Krista S. Crider, Owen Devine, Yan Ping Qi, Lorraine F. Yeung, Ahlia Sekkarie, Ibrahim Zaganjor, Eugene Wong, Charles E. Rose, Robert J. Berry

**Affiliations:** 1 Centers for Disease Control and Prevention, National Center on Birth Defects and Developmental Disabilities, Division of Congenital and Developmental Disorders, Atlanta, GA 30341, USA; owendevine2015@outlook.com (O.D.); irv7@cdc.gov (Y.P.Q.); lcy5@cdc.gov (L.F.Y.); cvr7@cdc.gov (C.E.R.); rjb1@cdc.gov (R.J.B.); 2 Karna, LLC, Atlanta, GA 30329, USA; 3 G²S Corporation, San Antonio, TX 78216, USA; 4 Oak Ridge Institute for Science and Education, Oak Ridge, TN 37831, USA; ahlia.sekkarie@emory.edu (A.S.); Ibrahim.Zaganjor@gmail.com (I.Z.); lgn8@cdc.gov (E.W.); 5 Doctoral Program in Nutrition Health Sciences, Laney Graduate School, Emory University, Atlanta, GA 30322, USA; 6 Department of Epidemiology, Gilling’s School of Global Public Health, University of North Carolina, Chapel Hill, NC 27599, USA

**Keywords:** folic acid, red blood cell folate, serum folate, plasma folate, fortification, supplementation, public health

## Abstract

The threshold for population-level optimal red blood cell (RBC) folate concentration among women of reproductive age for the prevention of neural tube defects has been estimated at 906 nmol/L; however, the dose-response relationship between folic acid intake and blood folate concentrations is uncharacterized. To estimate the magnitude of blood folate concentration increase in response to specific dosages of folic acid under steady-state conditions (as could be achieved with food fortification), a systematic review of the literature and meta-analysis was conducted. Of the 14,002 records we identified, 533 were selected for full-text review, and data were extracted from 108 articles. The steady-state concentrations (homeostasis) of both serum/plasma and RBC folate concentrations were estimated using a Bayesian meta-analytic approach and one-compartment physiologically-based pharmacokinetic models. RBC folate concentrations increased 1.78 fold (95% credible interval (CI): 1.66, 1.93) from baseline to steady-state at 375–570 µg folic acid/day, and it took a median of 36 weeks of folic acid intake (95% CI: 27, 52) to achieve steady-state RBC folate concentrations. Based on regression analysis, we estimate that serum/plasma folate concentrations increased 11.6% (95% CI: 8.4, 14.9) for every 100 µg/day folic acid intake. These results will help programs plan and monitor folic acid fortification programs.

## 1. Introduction

Neural tube defects (NTDs), such as anencephaly, encephalocele and spina bifida, often have devastating effects on infants and their families and can result in death or life-long disability. To help prevent NTDs, the Centers for Disease Control and Prevention (CDC) and the Institute of Medicine (IOM) recommend that women capable of becoming pregnant consume at least 400 µg of synthetic folic acid per day, in addition to having a diet rich in natural food folate [1,2]. This recommendation was informed by randomized controlled trials [3,4] and has been confirmed by fortification and supplementation programs showing that folic acid intake before and during early pregnancy prevents many NTDs [5,6,7,8,9,10,11]. To increase folic acid consumption in the U.S., the Food and Drug Administration (FDA) ruled that products labeled enriched (e.g., enriched cereal grain products) must contain 140 µg of folic acid per 100 g [12]. Since the full implementation of FDA-mandated folic acid fortification in 1998, NTD prevalence in the U.S. has dropped by 35%, representing approximately 1300 cases prevented and 500 million dollars saved each year [13,14]. At least 70 other countries also have legislation to mandate folic acid fortification a staple grain product although the effectiveness and coverage of most of these policies are not established nor widely monitored (http://www.ffinetwork.org/global_progress/). 

In order to monitor the population impact of folic acid fortification policies implementation, it is critical to have a biomarker that is associated with both the intake of folic acid and NTD risk to show that the folic acid fortified product is reaching the target population in amounts sufficient to decrease NTD risk. This allows more rapid evaluation without awaiting birth defects surveillance data that, while critical, takes many years to collect in sufficient numbers, is completely absent in many places around the world and can be affected by folate independent factors. Red blood cell (RBC) folate concentrations are associated with both folic acid intake and NTD risk; as RBC folate concentrations increase, NTD risk decreases up to 10 fold [15,16,17]. For the optimal prevention of NTDs, a population-level threshold for RBC folate concentration of >400 ng/mL (>906 nmol/L) was set by the World Health Organization (WHO) in 2015 [18]. In order to help implement the WHO recommendation in populations with different initial RBC folate concentrations, it is important to know how much blood folate concentrations increase in response to folic acid intake at different dosages at steady-state. Steady-state refers to the time when homoeostasis is re-established after initiation of folic acid intake and there are no additional changes in circulating plasma/serum or RBC folate concentration. To address these questions we conducted a systematic review and meta-analysis of folic acid intervention studies to estimate changes in RBC and serum/plasma folate concentrations at steady-state resulting from differing levels of folic acid intake. 

## 2. Materials and Methods 

### 2.1. Systematic Review

A systematic search of the literature was conducted by a CDC research librarian to include manuscripts published from 1 January 1980, through 31 May 2016 on Medline (OVID), Embase (OVID), CINAHL (Ebsco), Cochrane Library, PopLine, reference review and ClinicalTrials.gov. Studies were included if they had a baseline measurement and at least one follow-up measurement of serum/plasma and/or RBC folate concentrations (measured by the microbiologic assay) so that a change in blood folate concentrations could be reported in response to a known dose of folic acid. Exclusion criteria were: folic acid intake duration of <28 days (<4 weeks); supplementation of folate forms other than folic acid, folacin, or pteroylglutamic acid; routes of folic acid administration other than oral administration; children age <12 years (studies with some children age <12 years could be included as long as the study population was not predominantly <12 years); pregnant or lactating women; and unhealthy populations, defined as sample recruitment based on a health condition (including, but not limited to, conditions impacting folate absorption, such as intestinal malabsorption conditions, inflammatory bowel disease, and gastrointestinal surgery; alcohol abuse; smoking; cancer; chronic congestive heart failure; chronic renal failure; hemodialysis; chronic liver failure; parasitic infections and other chronic infectious disease; diabetes; polycystic ovarian syndrome; morbid obesity (body mass index ≥40 kg/m^2^); cognitive impairment; use of anti-folate medication; fertility treatment; or pre-existing conditions which may confound folic acid intake or biomarker response). In addition, studies were excluded if they did not use a microbiologic assay to measure RBC folate concentrations (serum/plasma data were included) or did not stratify blood folate concentrations by folic acid dose if multiple dosing levels of folic acid were used in the study. 

Three teams of two reviewers (KSC+YPQ, YPQ+IZ, and KSC+AS) screened and de-duplicated records to determine eligibility for full-text review followed by inclusion in modeling analyses. Relevant data were extracted from manuscripts which met the inclusion criteria after full-text review (Supplemental document: Protocol). Data were further checked for duplicate reporting from the same dataset (EW), and only results from the largest sample that met inclusion criteria from each data set were included in the final analysis (Table S1). Risk of bias (ROB) for each study was assessed by using a customized tool (Table S2). The tool was designed and piloted on three sets of manuscripts to assess replicability of findings (LFY, KSC). All manuscripts included in the analyses were assessed for risk of bias (LFY, KSC). (Table S3). 

### 2.2. Modeling Strategy

Our analysis goal was to estimate the ratio of the steady-state to baseline serum/plasma and RBC folate concentrations within folic acid dose strata. We will refer to this ratio as the steady-state ratio. Based on examination of the studies selected for inclusion in the analysis, folic acid doses were divided into the following strata based on the availability of studies of natural data clusters and gaps: 100–240, 375–570 and 800–1100 µg/day for studies reporting RBC folate concentrations and 50–250, 300–500, 563–714 and 800–1429 µg/day for studies reporting serum/plasma folate concentrations. 

To illustrate the modeling approach, let ***C***(**0**)***_ij_*** be the reported geometric mean serum/plasma or RBC folate concentration at baseline for the jth study in the i th folic acid dose strata (i.e., the geometric mean blood folate concentration at time 0). Similarly, let $C{\left(t\right)}_{ij}$ be the geometric mean folate concentration reported in the same study at week t. Our goal was to model the difference between the geometric mean blood folate concentration at time t (***C***(***t***)***_ij_***) and the geometric mean blood folate concentration at baseline (***C***(**0**)***_ij_***), in weeks, under an assumption of a constant uptake of folate, reflecting an approximate folic acid intake within each dose strata and first order kinetics for folate elimination, using the model

(1) $$\log\left(C{\left(t\right)}_{ij}\right)-\;\log\left(C{\left(0\right)}_{ij}\right)=\;log\left(\frac{C{\left(t\right)}_{ij}}{C{\left(0\right)}_{ij}}\right)\;=\;R{\left(t\right)}_{ij}$$

The assumptions of constant rate of folate uptake and elimination are reflected by use of the differential equation model

$$\frac{dR{\left(t\right)}_{ij}}{dt}\;=\;i_{i}\;-\;\lambda_{i}*t$$

where $\frac{dR{\left(t\right)}_{ij}}{dt}$ is the instantaneous change in the concentration ratio relative to baseline at time t, $i_{i}$ is the uptake rate of folate due to consumption of a constant folic acid dose, and $\lambda_{i}$ is the folate elimination rate. Solution of this differential equation, under the initial condition that at t=0, $R{\left(0\right)}_{ij}=0$ leads to the model,

(2) $$R{\left(t\right)}_{ij}\;=\;\frac{I_{i}}{\lambda_{i}}\;\left(1\;-\;e^{-\lambda_{i}*t}\right)$$

Note the subscript i on the parameters in Equation (2) indicating our assumption of constant uptake ($I_{i})$ and elimination ($\lambda_{i}$) for all studies in dose stratum i. 

We obtained observed values for $R{\left(t\right)}_{ij}\;$from the collection of studies within each folic acid dose stratum. To derive these values, we transformed measures of central tendency for the serum/plasma or RBC folate concentration reported for each measurement time as necessary to reflect the mean of the log transformed concentration values, that is, to an observed value for $log\left(C{\left(t\right)}_{ij}\right)$ [19,20]. Given these estimates, observed values for $R{\left(t\right)}_{ij}$ were derived for all measurement times using Equation (1). In addition, the corresponding standard error of these estimates, $std(R{\left(t\right)}_{ij}$) was also estimated for each time point within each study using methods outlined in [21]. 

To derive estimates of the input and elimination parameters, we used a Bayesian meta-analytic approach. However, we altered the model in Equation (2) to incorporate study-specific random effects reflecting both residual variation among studies and correlation across measurement time points [22]. To do this, we assumed that each $R{\left(t\right)}_{ij}$ is an independent random sample from a Normal distribution with mean

(3) $$\mu {\left(t\right)}_{ij}\;=\;\frac{I_{i}}{\lambda_{i}}\;\left(1\;-\;e^{-\lambda_{i}*t}\right)\;+\;u_{j}$$

and standard deviation equal to $std\left(R{\left(t\right)}_{ij}\right)$ In Equation (3), $u_{i}$ is a study random effect assumed to be a random variable distributed as *N*(0, $\sigma_{i}$). To complete the specification of the model, we assumed uniform prior distributions for both $I_{i}$ and $\lambda_{i}$ with bounds defined as 0 to 10 and 0 to 5, respectively. In addition, we assumed a uniform (0, 100) prior for the standard error of the study random effects, $\sigma_{i},$ within each dose stratum [23].

Because our goal is to estimate the ratio of the steady-state to baseline concentration for each dose group, which we will refer to as $SSR_{i}$, the estimand of interest derived from the model in Equation (3) is

(4) $$SSR_{i}\;=\;\frac{I_{i}}{\lambda_{i}}$$

that is, the asymptotic value for $R{\left(t\right)}_{ij}$.

Parameters in the model given in Equation (3) were derived using Markov Chain Monte Carlo (MCMC) methods [23]. Models were fit separately within each dose stratum. Two sampling chains were used with highly divergent starting values to enable graphical assessment of convergence to the posterior distribution. Each sampling chain was run to 100,000 samples with the first 50,000 samples discarded as burn-in. In addition, only every fifth sample after the 50,000 burn-in was retained to reduce autocorrelation among the samples used to estimate the posterior distributions. As a result, estimates of the posterior distribution of the steady-state ratio, $SSR_{i}$ in Equation (4), were based on 20,000 samples. These distributions were summarized using the median of the 20,000 samples with uncertainty reflected by a 95% credible interval defined as the range separating the 2.5th and 97.5th percentiles of the samples.

To enable comparison of the modeled values of $R{\left(t\right)}_{ij}$ to those observed in the included studies, we also produced samples from the posterior predictive distribution for $R{\left(t\right)}_{ij}\;$for each week from 0 to the largest t reported among the studies in each dose strata [23]. To do this, we utilized the posterior samples for $I_{i}$ and $\lambda_{i}$ to generate predicted values for the log concentration within dose strata i at time t to baseline ratio, ${\hat{R\left(t\right)}}_{i}$, such that.

(5) $${\hat{R\left(t\right)}}_{i}\;\sim \;N(\mu {\left(t\right)}_{i},\;\sigma_{i}^{2})$$

where

$$\mu {\left(t\right)}_{i}\;=\;\frac{I_{i}}{\lambda_{i}}\;\left(1\;-\;e^{-\lambda_{i}*t}\right)$$

Given posterior samples for $\mu {\left(t\right)}_{i}$ and $\sigma_{i}$, 20,000 samples for ${\hat{R\left(t\right)}}_{i}$ were estimated for each time point in every dose strata under the distributional assumption given in Equation (5). Both the generated values, ${\hat{R\left(t\right)}}_{i}$, and the observed study-specific ratios, $R{\left(t\right)}_{ij}$, were then exponentiated to the scale of absolute percentage increase over baseline to facilitate interpretation. Uncertainty in the estimated ratios at each time point was summarized using a 95% posterior predictive interval (PI) defined by the 2.5th and 97.5th percentiles of the collection of estimates. This interval can be interpreted as the uncertainty one would expect, under our assumed model, in the ratio value at each time point in a hypothetical future study having a folic acid dose similar to that in dose stratum i.

### 2.3. Estimation of the Time to Steady State

In addition to developing estimates of the steady-state to baseline ratios for each folic acid dose stratum, we also estimated the time, in weeks, it took to reach that steady state. However, due to the asymptotic structure of the model in Equation (3), there is no analytic solution for this steady-state time. As a result, we approximated the time of reaching steady-state concentration by evaluating the 20,000 posterior ratio estimates and setting time to steady-state as the first time in which the estimated ratio to baseline concentration was within 1% of the exponentiated steady-state ratio estimate given in Equation (4). These estimates were summarized using the median and a 95% credible interval was defined by the 2.5th and 97.5th percentiles of the posterior samples.

### 2.4. Sensitivity Analyses

Further analyses were conducted for each dose stratum to assess the impact of ROB (low/medium vs. high), age (≤50 vs. >50 years), and baseline folate concentrations (<median vs. ≥median) on the estimates of the steady-state ratio. For the ROB assessment, studies within each dose stratum were subdivided into either high or low/medium categories based on the assigned ROB value for the study. Low and medium ROB studies were combined into one stratum due to the small number of studies given a low ROB score. Steady-state ratio estimates were then estimated under the model in Equation (3) within each variable (e.g., age) and dose stratum and compared across variable categories.

For the age assessment, studies in each folic acid dose stratum were subdivided into categories reflecting the age of study participants with one category defined as age less than or equal to 50 and the other by age greater than 50, since women of reproductive age are often defined as ≤49 years and this was a natural break in the data. 

We used a similar stratification approach to assess the potential impact of baseline folate concentration on the estimated steady-state ratio. In this case, a given study was assigned to a low baseline concentration category if the geometric mean baseline concentration reported in that study was less than the median geometric mean baseline among all studies in that folic acid dose strata. Alternatively, a study was categorized as having a high baseline concentration if the reported baseline geometric mean concentration in that study was greater than or equal to the median strata-specific baseline geometric mean.

### 2.5. Serum/Plasma Folate

A large number of studies reporting serum/plasma folate concentration were available to enable estimation of the steady-state ratio across multiple folic acid dose strata. In this approach, we let ${\hat{SSR}}_{i}$ be the median and $\gamma_{i}$ be the standard deviation of the estimated posterior distribution for the steady-state ratio for dose group i derived using the MCMC approach described above. We assume that

$${\hat{SSR}}_{i}\;\sim \;N\left(\beta_{0}\;+\;\beta_{1}*d_{i},\;\gamma_{i}^{2}\right)$$

where $\beta_{0}$ and $\beta_{1}$ are parameters of the linear regression of the steady-state ratio on dose and $d_{i}$ is the mean daily folic acid dose among all studies in dose strata i. To complete specification of the model, we assumed non-informative Normal priors for both $\beta_{0}$ and $\beta_{1}$. Estimates for the posteriors of the model parameters were derived using the same MCMC approach as was used to estimate the steady-state ratios. Recalling the definition of the steady-state ratios as

$$SSR_{i}\;=\;log\left(\frac{C{\left(SS\right)}_{i}}{C{\left(0\right)}_{i}}\right),$$

where $C{\left(SS\right)}_{i}$ is the serum/plasma folate concentration at steady-state in dose group i, then $e^{\beta_{1}}$ provides an estimator for the percentage increase in the steady-state serum/plasma concentration per unit of folic acid intake. Using this result, we estimated the posterior distribution for $e^{\beta_{1}*400}$ corresponding to the percentage increase in the steady-state serum/plasma concentration resulting from an increase of 400 µg/day in folic acid intake. Again, the posterior distribution estimates are summarized using the median values and 95% credible intervals.

## 3. Results

**Figure 1:**
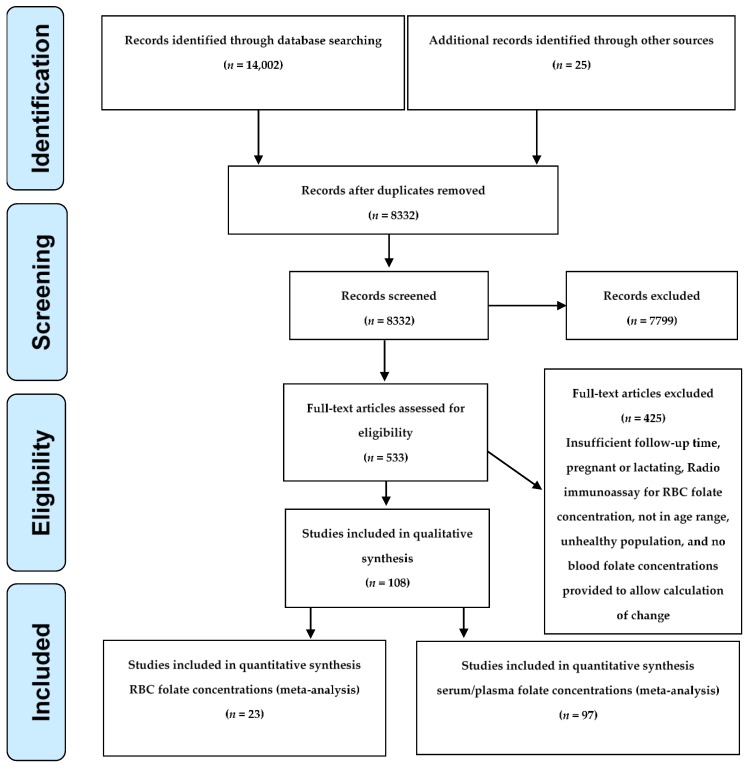
PRISMA Flow Diagram. RBC: red blood cell.

The initial literature search yielded 14,002 abstracts and 25 additional ones from other sources. After de-duplication and review, we identified 533 articles for full-text review. Of these, we included 108 articles in the qualitative synthesis (Figure 1, Table S1). In the end, the criteria for the meta-analysis were met by 23 articles for RBC folate and by 97 articles for serum/plasma folate. Individual articles could include both RBC and serum/plasma folate and/or multiple dosages (Table S1). 

For the 17 studies that included dosages of 375–570 µg/day of folic acid, the estimated ratio of steady-state to baseline of RBC folate concentrations was 1.78 (95% CI: 1.66, 1.93) and it took 36 weeks (95% CI: 27, 52) to achieve steady-state (Table 1, Figure S1). There were insufficient studies for the models to converge for other dosages of folic acid. In the sensitivity analyses (Table 2), we found that studies with a high ROB had a lower ratio of baseline to steady-state (1.50; 95% CI: 1.35, 1.66) than those studies with low or medium risk of bias (1.81; 95% CI: 1.66, 2.00). In addition, studies with lower initial RBC folate concentrations (<615 nmol/L) had a higher ratio of steady-state to baseline (1.85; 95% CI: 1.70, 2.02) compared to studies with higher initial RBC folate concentrations (≥615 nmol/L; 1.43 fold increase; 95% CI: 1.32, 1.58). Age could not be assessed in these sensitivity analyses because all of the studies with RBC folate concentrations were in populations whose members were less than 50 years of age. We also examined the effects of assay type for serum/plasma concentrations and found that microbiologic assay vs. non-microbiologic assay studies for the 300–500 groups had similar estimates (microbiologic: 2.12 fold increase from baseline to steady-state (95% CI: 91.79, 2.52; 95% PI: 1.00, 4.59), 18 studies (*n* = 30), time to asymptote 11 weeks (95% CI: 6, 15), and max follow up weeks = 48 vs. non-microbiologic: 1.96 fold increase from baseline to steady-state (95% CI: 1.73, 2.22; 95% PI: 0.91, 4.16), 35 studies (*n* = 49), time to asymptote 15 weeks (95% CI: 10, 23), and max follow up weeks = 120). 

**Figure 2:**
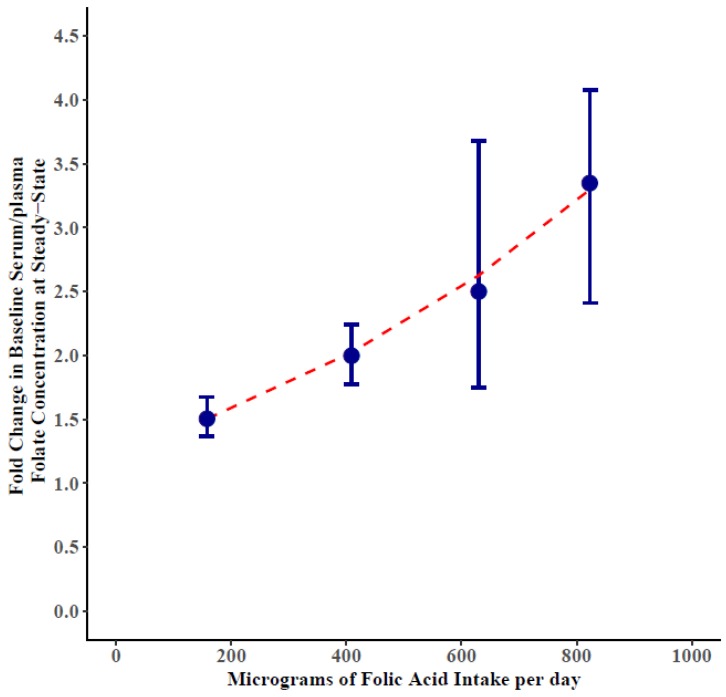
Folic acid intake and change in ratio of steady-state to baseline serum/plasma folate concentrations by folic acid dosage. The blue dot is the point estimate of the folate change in serum/plasma folate concentration plotted at steady-state of the median folic acid intake for the range (50–250, 300–500, 563–714, and 800–1429 μg/day) and 95% Credible Intervals (CI) error bars. The slope of the regression model to estimate concentration changes across the entire range of dosages is shown. The ratio of steady-state to baseline serum/plasma folate concentration increased by 11.6% (95% CI: 8.4, 14.9) for every increase in 100 µg/day of folic acid dose.

For the 53 studies that included dosages of 300–500 µg/day of folic acid (Table 1), the estimated ratio of steady-state to baseline of serum/plasma folate concentrations was 2.00 (95% CI: 1.81, 2.21) and it took 13 weeks (95% CI: 10, 16) to achieve steady-state. For the 35 studies that included dosages of 50–250 µg/day of folic acid (Table 2), the estimated ratio of steady-state to baseline of serum/plasma folate concentrations was 1.50 (95% CI: 1.40, 1.62). For the 25 studies that included dosages of 800–1429 µg/day of folic acid (Table 2), the estimated ratio of steady-state to baseline of serum/plasma folate concentrations was 3.35 (95% CI: 2.76, 4.05). The 563–714 µg/day folic acid dosage group did not converge for sensitivity analyses. Because there were enough studies for each of these dose ranges (Figure S2), we could also fit a regression model to estimate concentration changes across the entire range of dosages. With this model we found that the ratio of steady-state to baseline serum/plasma folate concentration increased by 11.6% (95% CI: 8.4, 14.9) for every increase in 100 µg/day of folic acid dose (Figure 2). 

In the sensitivity analysis of serum/plasma (Table 2), we found that there was no consistent impact of ROB or age on the ratios. However, for each dosage group the lower initial serum/plasma folate concentrations had higher estimated ratios of steady-state to baseline when compared to the higher initial serum/plasma folate concentrations. 

## 4. Discussion

This is the first meta-analysis to our knowledge to estimate how much blood folate concentrations increase in response to folic acid intake under steady-state conditions. We found that when folic acid in the range that included the recommended daily intake for NTD prevention (400 µg) was consumed, RBC folate concentrations increased over baseline by 1.78 fold and steady-state was reached by 36 weeks. An example of how these findings can be interpreted is that in a population with a median RBC folate concentration of 600 nmol/L, a folic acid fortification program targeting the recommended intake of 400 µg/day could increase the median population RBC folate concentration to about 1068 nmol/L (95% CI: 996, 1158) after 9 months of intervention (assuming the intervention reached the population and was consumed regularly). Increasing RBC folate concentrations would result in a decrease in NTD risk from a median risk of about 19 per 10,000 to about 7 per 10,000 [17]. The analyses reported here on the estimated fold change in RBC folate concentrations and variance in response to folic acid intake would help to inform the additional folic acid intake required among populations of women whose RBC folate concentrations are below the threshold for optimal NTD prevention. As more is not always better, alternatively, these analyses can also help us avoid setting folic acid intakes that would result in high RBC folate concentrations with limited additional benefit for NTD prevention [24]. Unfortunately, there were few studies of lower and higher folic acid doses that reported RBC folate concentrations; thus, we were unable to estimate steady-state increases at other doses or as a per unit increase over the usual intake range. Additional long-term studies are needed of to define the relation between lower folic acid dosages and changes in RBC folate concentrations to reflect appropriate folic acid fortification levels for populations with moderate baseline folate concentrations. 

By comparison, when the recommended dosage of folic acid was consumed, serum/plasma concentrations increased by 2 fold and steady-state was reached by 13 weeks. For serum/plasma folate, as folic acid intake dosage increased, the ratio of steady-state to baseline concentration increased about 12% per 100 µg/day increase. A previous analysis of biomarker dose-response to folic acid intake restricted to randomized controlled trials found less change in both serum/plasma as well as RBC folate concentrations due to differences in study design. Primarily their approach grouped shorter interventions with longer interventions [25], which would lead to an underestimation of actual increase due to the many weeks it takes to achieve steady-state. In addition, our study used PBPK (physiologically based pharmacokinetic) models which describe folate uptake and elimination to estimate the steady-state concentrations (homeostasis) enabling a more representative model of the long-term impact of folic acid daily intake from fortification programs on the folate status biomarkers. 

### 4.1. Implications for Implementing the World Health Organization (WHO) Guidelines

Measurement of population-based blood folate concentrations and surveillance of birth defects, before and during the implementation of folic acid fortification programs, are critical components of the WHO guidelines and reaching the optimal RBC folate concentration threshold of >906 nmol/L [18]. For those conducting evaluations of folic acid intervention policies, our estimate of 9 months for RBC folate concentrations to achieve steady-state provides important information for the planning and implementation of blood biomarker surveillance monitoring, including allowing for sufficient time for folic acid to be added to the staple that is being fortified and for the population consuming the product to reach steady state. Sampling too early after the intervention will likely underestimate the changes in blood folate concentrations. 

There are complex considerations for appropriate choice of folate status indicator. RBC folate concentrations and serum/plasma folate concentrations are both biomarkers of folate status, although only RBC folate concentrations have been linked to NTD risk to date and have a defined optimal threshold for NTD prevention by WHO [16,17,18,26]. In general, RBC folate concentrations are long-term markers of folate status over the previous months of folate intake that has been processed into the cells, while serum/plasma folate concentrations are generally considered short-term markers [27]. The differences in both the time (36 weeks RBC vs. 13 weeks serum/plasma) and moderate differences in magnitude of change in baseline to steady-state concentration ratio (1.78 RBC vs. 2.0 serum/plasma fold change) underscore the fact that these two measures, although both responsive to folic acid intake, are different biological biomarkers. RBC folate concentrations are the only folate biomarker directly tied to NTD risk [16,17,26]. 

### 4.2. Implications for Individual Women

The results presented here are intended to inform population-based folic acid fortification programs, not the clinical care of individual women. The current recommendation for women capable of becoming pregnant by CDC and the Institute of Medicine is that all women capable of becoming pregnant consume at least 400 µg/day of folic acid from supplements, fortified food or a combination, in addition to a diet rich in natural food folate [1,2]. The time it takes to reach and surpass the optimal RBC folate concentration threshold is a function of the baseline concentration and the intake dose. We found that it takes about 9 months for RBC folate concentrations to reach steady-state concentrations for those consuming the 375–570 µg/day dose. However, this does not imply that women must start taking folic acid 9 months before pregnancy to benefit; rather, 9 months was the average time for RBC folate concentrations in the blood to stabilize. Other evidence shows that RBC folate concentrations begin to increase quickly following initiation of intake [15] and NTD risk decreases as RBC folate concentration increases [18]. Thus, any intake is likely beneficial, but longer duration of intake would be associated with the lowest risk of NTDs. It is important, nevertheless, that women capable of becoming pregnant regularly consume folic acid in recommended dosages because 45% of pregnancies in the United States are unplanned [28] and pregnancy is typically not recognized until after the time period relevant to closure of the neural tube (i.e., the first four weeks post-conception). If folic acid consumption is only initiated after a woman learns she is pregnant, it is generally too late to prevent NTDs. 

### 4.3. Limitations

All meta-analyses are subject to the limitation of the data that are included. The optimal RBC folate concentration for NTD prevention threshold is based on the microbiologic assay calibrated with folic acid [17,18]. Unfortunately not many studies measure RBC folate concentrations and even fewer use assays that are free of bias in quantitation of the folate forms (microbiologic assay) [29,30], thus limiting the number of eligible studies. In addition, there are challenges of producing similar results even when using the same assay due to calibration considerations [30]. All meta-analysis combining data from different groups and assay should be interpreted with caution due to these issues. Unfortunately, there were not enough data to model the impact of lower (or higher) dosages for folic acid exposure on RBC folate concentrations. This is a significant limitation for settings with some limited fortification or existing moderate (but not quite optimal) RBC folate concentrations as an additional 400 µg/day would move RBC folate concentrations above what is needed for optimal prevention. Additional studies using lower dosage of folic acid and with RBC folate measured with the microbiologic assay are needed. 

In addition there are a limited number of studies that extend to a year or beyond at any dosage (Table S1). The limited amounts of data are reflected in the width of both of the uncertainty measure presented, the 95% credible intervals (uncertainty in this existing data) and the 95% posterior predictive intervals (uncertainty associated with adding new studies) (Table 1 and Table 2). As suggested we have kept data unadjusted [29] and then modeled the change in blood folates from baseline to help mitigate any absolute differences in the assay results between laboratories [30]. The non-microbiologic assays were not used for the analyses on the association of folic acid intake and changes in RBC folate concentrations as the results are biased showing differential affinity for folate forms and inaccurate individual results [29,30]. The issues of comparability of absolute RBC folate concentrations between version of the microbiologic assay and the various calibrators and methods are critical for appropriate use of the any data to be compared to a standard threshold for insufficiency (NTD risk) or deficiency (anemia risk) [29,30]. As part of a sensitivity analysis we did not find any differences between results for the microbiologic vs. non-microbiologic for serum/plasma folate concentrations, confirming what we had seen in previous meta-analyses, which is likely due to the predominance of 5’ MTHF in circulation [31,32]. The extended amount of time to reach steady-state of RBC folate concentrations with folic acid supplementation is also consistent with results from a study of supplementation with (6S)-5-methyltrahydrofolate in which steady-state was achieved between 6 and 12 months of supplementation [33]. 

Although it would be ideal to only group studies with the exact same dosage of folic acid and 100% compliance, grouping similar dosages was necessary for statistical power. Importantly, we have previously found that women of reproductive age in the United States who have RBC folate concentrations in the optimal range (906–1500 nmol/L) have estimated intakes of the equivalent of 350 µg/day folic acid (25th –75thile: 285–426) [17] similar to those modeled together in our analysis. In any free-living population there will be variation in actual daily folic acid intake, even in populations exposed to fortified food, due to differences in supplement use and consumption of fortified foods. The variance in the models reflect this lack of precision and the limitations of the meta-analyses (Table 1 and Table 2). 

Our sensitivity analysis showed that studies’ risk of bias and age had limited impact on our main findings. However, initial folate concentration was impactful for both RBC and serum/plasma folate, with lower baseline concentrations being associated with greater ratios of steady-state to baseline. Unfortunately, there were not enough studies for a finer-grained examination of initial folate concentrations and impact of different initial concentrations beyond above and below medians. In addition our meta-analysis modeled change. 

This analysis used data from intervention studies, generally supplementation trials. For the results to more closely represent real-world settings, it would be ideal to have fortification studies that made repeated measures across time of folic acid intake and blood folate concentrations, with a consistent blood folate assay. However, such studies do not exist in the literature at this time. A previous study showed that a folic acid dosage of 400 µg/day and 100 µg/day produce the same change in RBC and plasma folate if given in 4 equal portions (similar to a fortification dosage) vs. in one supplement [15], although with fortification there is likely more variation in dosages on a day to day basis because of variation in the quantity of fortified foods consumed. The impact of the fortification vehicle (e.g., wheat, corn, salt) on usual daily intake is important to account for in fortification policy implementation as there are differences in food patterns that a unique to each setting as well as day to day and season to season. 

## 5. Conclusions

Our systematic review and meta-analysis found that after initiation of folic acid intake, both RBC folate and serum/plasma folate concentrations increase. At 375–570 µg folic acid/day, RBC folate concentrations increased 1.78 fold (95%CI: 1.66, 1.93) from baseline to steady-state, over a median of 36 weeks (95% CI: 27, 52). For every 100 µg/day folic acid intake, serum/plasma folate concentrations increased 11.6% (95% CI: 8.4, 14.9) from baseline to steady-state, over a median of 13 weeks (95% CI: 10, 16). These results can inform how much additional folic acid intake is needed among populations of women whose RBC folate concentrations are below the optimal threshold. 

## Figures and Tables

**Table 1:** Estimated ratio of steady-state to baseline red blood cell (RBC) and serum/plasma folate concentrations and estimated time to reach steady state.

	Folic Acid Dose (µg/day)	Number of Studies Used for Each Analysis *	Maximum Follow-up in Weeks in Each Analysis	Estimated Ratio of Steady-state to Baseline Folate Concentrations (95% Credible Interval)	95% Posterior Predictive Interval ^ŧ^	Estimated Weeks to Reach Steady-state (95% Credible Interval)
RBC folate concentrations						
	375–570	17	48	1.78 (1.66, 1.93)	1.37, 2.34	36 (27, 52)
Serum/Plasma folate concentrations						
	50–250	35	48	1.50 (1.40, 1.62)	1.03, 2.20	8 (3, 12)
	300–500	53	120	2.00 (1.81, 2.21)	0.96, 4.18	13 (10, 16)
	563–714 ˠ	7	24	2.50 (1.76, 3.64)	0.80, 7.94	11 (7, 17)
	800–1429	25	144	3.35 (2.76, 4.05)	1.25, 9.02	15 (11, 20)

* Individual manuscripts could include multiple doses ^ŧ^ Uncertainty in the estimated ratios at each time point was summarized using a 95% posterior predictive interval defined by the 2.5th and 97.5th percentiles of the collection of estimates. This interval can be interpreted as the uncertainty one would expect, under our assumed model, in the ratio value at each time point in a hypothetical future study having a folic acid dose similar to that in dose stratum *i*. ˠ Too few studies to include this group in sensitivity analysis.

**Table 2:** Estimated ratio of steady-state to baseline red blood cell (RBC) and serum/plasma folate concentrations by initial folate concentration and risk of bias.

	Folic Acid Dose (µg/day)	Strata	Number of Studies *	Maximum Follow-up in Weeks *	Estimated Ratio of Steady-state to Baseline Folate Concentration	95% Credible Interval	95% Posterior Predictive Interval ^ŧ^
**RBC folate concentrations**							
	375–570						
		**Risk of Bias**					
		High	9	48	1.50	1.35, 1.66	1.15, 1.95
		Low, Medium	8	40	1.81	1.66, 2.00	1.33, 2.49
		**RBC folate initial values (nmol/L)**					
		<615	9	24	1.85	1.70, 2.02	1.38, 2.52
		≥615	8	48	1.43	1.32, 1.58	1.18, 1.77
**Serum/Plasma folate concentrations**							
		**Risk of Bias**					
	50–250	High	21	24	1.40	1.27, 1.53	0.95, 2.06
		Low, Medium	14	48	1.62	1.46, 1.80	1.09, 2.42
	300–500						
		High	33	120	2.19	1.85, 2.58	0.94, 5.20
		Low, Medium	20	40	1.89	1.66, 2.18	0.99, 3.64
	800–1429	High	12	24	3.19	2.44, 5.21	1.09, 10.81
		Medium	13	144	3.35	2.44, 4.44	1.04, 10.42
		**Serum/plasma initial values (nmol/L)**					
	50–250	<15.6	17	48	1.66	1.49, 1.85	1.09, 2.55
		≥15.6	18	48	1.37	1.27, 1.50	1.00, 1.87
							
	300–500	<16.4	21	120	2.52	2.06, 2.98	1.07, 5.89
		≥16.4	32	48	1.64	1.49, 1.82	0.99, 2.70
							
	800–1429	<12.5	12	144	4.41	3.78, 4.93	2.82, 6.66
		≥12.5	13	30	2.64	2.06, 3.57	0.94, 7.95
		**Age years**					
	50–250	≤50	22	48	1.46	1.32, 1.62	0.93, 2.28
		>50	13	48	1.57	1.41, 1.75	1.15, 2.14
							
	300–500	≤50	32	48	2.01	1.75, 2.30	0.91, 4.36
		>50	21	120	2.02	1.74, 2.35	0.96, 4.22
							
	800–1429	≤50	10	30	3.53	2.59, 4.99	1.27, 9.97
		>50	15	144	3.04	2.33, 4.08	0.92, 10.07

* Individual manuscripts could include multiple doses ^ŧ^ Uncertainty in the estimated ratios at each time point was summarized using a 95% posterior predictive interval defined by the 2.5th and 97.5th percentiles of the collection of estimates. This interval can be interpreted as the uncertainty one would expect, under our assumed model, in the ratio value at each time point in a hypothetical future study having a folic acid dose similar to that in dose stratum *i*.

## Supplementary Materials

The following are available online at http://www.mdpi.com/2072-6643/11/1/71/s1, Table S1. Studies that met inclusion criteria and are included in the qualitative analysis and eligible for the meta-analysis; Table S2: Risk of bias (ROB) assessment tool questions by study design, as adapted from the Item Bank for Assessment of Risk of Bias and Precision for Observational Studies; Table S3: Summary risk of bias tables for randomized controlled trials using Cochrane tool and for non-randomized controlled trials and observational studies using adapted RTI item bank. Protocol: Folic acid supplementation and blood folate concentration—a systematic review and meta-analysis; Figure S1: Fold change in serum/plasma folate concentrations from baseline over time in weeks for each study extracted; Figure S2: Fold change in red blood cell (RBC) folate concentrations from baseline over time in weeks for each study extracted.
