# Supplementary material for: Systematic Review and Bayesian Meta-analysis of the Dose-response Relationship between Folic Acid Intake and Changes in Blood Folate Concentrations
Source: Nutrients. 2019 Jan 2;11(1):71. doi: 10.3390/nu11010071 (PMC6356991; doi:10.3390/nu11010071)
Supplement: Supplementary file 1 [file nutrients-11-00071-s001.pdf]

Table S1. Studies that met inclusion criteria and are included in the qualitative analysis and eligible for the meta-analysis

Reference	Type of Study	Population Characteristics[3]	Intervention	Biomarker	Blood assay method[4]	Timing of exposure *Level/type of folate*	Serum/Plasma folate (nmol/L)	RBC folate (nmol/L)	Dietary folate intake (µg/d)
**Aarabi, M. 2015 [1]**	Dietary intervention trial	30 infertile men<br>Age: 37.9± 1.3[5]<br>Montreal, Canada<br>2009-2011	5mg of FA/d for 6 mo	RBC folate	Microbiologic - *L. casei*	**Baseline:**<br>5000 µg FA (n=28)	Unavailable	273 ± 24[6]	Unavailable
						**Intervention: 24 wks**<br>5000 µg FA (n=19)	Unavailable	488 ± 35[6]	Unavailable
**Adank, C. 2003 [2]**	Randomized control trial	138 healthy women<br>Age: 18-40[7]<br>Dunedin, New Zealand<br>July - Oct 2000	**Intervention:** FA supplement feeding study (12 wks)<br>Dietary questionnaire at baseline only	Plasma and RBC folate	Microbiologic - *L. casei*	**Baseline:**<br>400 µg/d FA: (n=36)<br>2800 µg/wk FA: (n=41)	19.7 ± 10.0[5]<br>22.9 ± 13.0[5]	645 ± 211[5]<br>624 ± 177[5]	223 (201, 246)[8]<br>260 (227, 292)[8]
						**Intervention: 12 wks**<br>400 µg/d FA: (n=36)<br>2800 µg/wk FA: (n=41)	Unavailable	1095 ± 295[5]<br>913 ± 225[5]	Unavailable
**Amatayakul, K. 1984 [3]**	Randomized controlled trial	67 healthy nonpregnant women<br>Age: 18-35[7]	300 µg FA/d or placebo for 1 yr	Serum and RBC folate	Microbiologic - *L. casei*	**Baseline:**<br>300 µg FA/d (n=33)	24.3 ± 9.1[5,9]	706 ± 199.8[5,9]	Unavailable
						**Intervention: 15 wks**<br>300 µg FA/d (n=33)	37.5 ± 10.1[5,9]	944.3 ± 217.9[5,9]	Unavailable
						**Intervention: 27 wks**<br>300 µg FA/d (n=33)	37.7 ± 10.0[5,9]	957.9 ± 208.8[5,9]	Unavailable
						**Intervention: 48 wks**<br>300 µg FA/d (n=33)	38.4 ± 11.7[5,9]	1023.8 ± 227.0[5,9]	Unavailable
**Anderson, C. 2010 [4]**	Randomized control trial	107 healthy males and females<br>Age: 60-90[7]	100 µg FA/d, 400 µg FA/d, 1000 µg FA/d, or 2000 µg FA/d for 6 wks	Serum folate	Radio-protein binding Quantaphase II (Bio-Rad Laboratories, Hercules, California)	**Baseline:**<br>100 µg/d FA: (n=27)<br>400 µg/d FA: (n=27)<br>1000 µg/d FA: (n=26)<br>2000 µg/d FA: (n=27)	10.9 (8.4 - 15.2)[8,9]<br>15.2 (8.4 - 18.4)[8,9]<br>13.6 (9.3 - 17.5)[8,9]<br>15.0 (12.3 - 17.9)[8,9]	Unavailable	244 (195-309)[8]<br>289 (221-341)[8]<br>281 (206-364)[8]<br>302 (184-388)[8]
						**Intervention: 6 wks**<br>100 µg FA/d (n=27)<br>400 µg FA/d (n=27)<br>1000 µg FA/d (n=26)<br>2000 µg FA/d (n=27)	15.9 (12.3 - 21.3)[8,9]<br>28.1 (21.8 - 37.9)[8,9]<br>36.5 (28.1 - 74.0)[8,9]<br>37.3 (24.3 - 148.7)[8,9]	Unavailable	Unavailable

**Anderson, C. 2013 [5]**	Randomized, double blind, crossover, control trial	235 randomized male and females; 142 anaylzed<br>Age: 18-69[7]<br>Seattle, WA, 1998-2001	0, 200, or 400 µg FA/d<br><br>2 12 wks supplementation intervention with a 30 wk washout period in between (AWO)	Serum and RBC folate	***From Author directly:***<br><br>***Abbott IMx folate assay (ion capture assay) for Serum folate and RBC folate. In May 1999 when the Abbott Labs no longer used the IMx we switched to using the Beckman Access method***	**Baseline:**			
						**Group B:** 200 µg first (n=34)	31.3 ± 1.04[6,9]	n/a[10]	Unavailable
						**Group C:** 200 µg first (n=34)	32.5 ± 0.86[6,9]		
						**Group A:** 200 µg AWO (n=42)	26.8 ± 0.95[6,9]		
						**Group D:** 200 µg AWO (n=32)	25.0 ± 1.11[6,9]		
						**Group D:** 400 µg first (n=32)	31.6 ± 1.11[6,9]		
						**Group C:** 400 µg AWO (n=34)	25.7 ± 1.07[6,9]		
						**Intervention: 6wks**			
						**Group B:** 200 µg first (n=34)	33.8 ± 1.04[6,9]	n/a[10]	Unavailable
						**Group C**: 200 µg first (n=34)	34.1 ± 0.75[6,9]		
						**Group A**: 200 µg AWO (n=42)	35.2 ± 1.73[6,9]		
						**Group D:** 200 µg AWO (n=32)	31.1 ± 1.61[6,9]		
						**Group D:** 400 µg first (n=32)	35.9 ± 1.97[6,9]		
						**Group C**: 400 µg AWO (n=34)	40.0 ± 4.63[6,9]		
						**Intervention: 12 wks**			
						**Group B**: 200 µg first (n=34)	33.8 ± 0.86[6,9]	n/a[10]	Unavailable
						**Group C:** 200 µg first (n=34)	32.9 ± 0.77[6,9]		
						**Group A**: 200 µg AWO (n=42)	36.8 ± 3.04[6,9]		
						**Group D:** 200 µg AWO (n=32)	32.2 ± 1.63[6,9]		
						**Group D:** 400 µg first (n=32)	36.6 ± 1.63[6,9]		
						**Group C:** 400 µg AWO (n=34)	45.4 ± 3.84[6,9]		
**Ashfield-Watt, P. 2001 [6]**	Randomized control trial	45 participants<br>Age: 18-70[7]	250 µg FA/d for 3 months	Plasma folate	Competitive protein binding assay, Abbott IMX methods	**Baseline:**<br>250 µg FA (n=50)	12.7 ± 4.5[5,9]	Unavailable	Unavailable
						**Intervention: 3 mo**<br>250 µg FA (n=45)	19.5 ± 8.4[5,9]	Unavailable	Unavailable
**Ashfield-Watt, P. 2002 [7]**	Crossover, dietary intervention trial	126 healthy subjects<br>Age: 20-63[7]	400 µg FA/d for 4 months	Plasma folate	Competitive protein binding assay (Abbott IMX)	**Baseline:**<br>352 µg FA/d (n=126)	17.7 ± 7.7[5]	Unavailable	214 ± 70[5]
						**Intervention: 4 mo**<br>352 µg FA/d (n=108)	31.7 ± 16.5[5]	Unavailable	201 ± 63[5]
**Baro, L. 2003 [8]**	Dietary intervention trial	30 residents in Spain<br>Age: 20-45[7]	drank 500 ml/d of semi-skimmed milk for 4 weeks (depletion) followed by 8 weeks of enriched semi-skimmed milk at 500 ml/d (150 µg FA/d)	Plasma folate	Immunoassay, SimulTRANC-SNB Radioassay Kit (ICN Pharmaceuticals, USA)	**Baseline:**<br>150 µg FA/d (n=30)	7.6 ± 0.8[6,9]	Unavailable	Unavailable
						**Intervention: 4 wks**<br>150 µg FA/d (n=30)	12.7 ± 1.0[6,9]	Unavailable	Unavailable

						**Intervention: 8 wks**<br>150 µg FA/d (n=30)	13.8 ± 1.0[6]	Unavailable	Unavailable
**Baro, L. 2004 [9]**	Dietary intervention trial	31 healthy non-pregnant women<br>Age: 21 – 37[7]<br>Guatemala	400 µg FA/d from fortified dairy products for 8 wks	Plasma folate	Immunoassay, SimulTRANC-SNB Radioassay Kit (ICN Pharmaceuticals, USA)	**Baseline:**<br>400 µg/d FA (n=31)	8.6 ± 0.9[6]	Unavailable	Unavailable
						**Intervention: 8 weeks**<br>400 µg/d FA (n=31)	21.0 ± 1.4[6]	Unavailable	Unavailable
**Basoglu, C. 2009 [10]**	Randomized controlled trial	35 patients with depression<br>Age: 18-66[7]	2500 µg FA/d for 6wks +Escitalopram	Serum folate	Electro-chemoluminescence immunoassay	**Baseline:**<br>2500 µg FA/d (n=20) + Escitalopram	12.3 (5.7, 23.2)[9,11]	Unavailable	Unavailable
						**Intervention: 6 wks**<br>2500 µg FA/d (n=20) + Escitalopram	15.9 (8.4, 45.4)[9,11]	Unavailable	Unavailable
**Biswas, A. 2009 [11]**	Dietary intervention trial	24 stroke patients<br>Age: under 40<br>Northern India	5000 µg FA/d for 12 wks	Serum folate	Radioimmunoassay, Immulite (Diagnostic Product Corporation, Erlangen, Germany)	**Baseline:**<br>5000 µg FA/d (n=24)	11.2 (8.1, 14.2)[11]	Unavailable	Unavailable
						**Intervention: 12 wks**<br>**50**00 µg FA/d (n=24)	11 (8.2, 14.2)[11]	Unavailable	Unavailable
**Bramswig, S. 2009 [12]**	Double-blind, placebo controlled intervention	46 healthy females<br>Age:18-35[7]<br>Germany	Intervention: daily multivitamin 800 µg FA for 16 weeks	RBC and Plasma folate	Microbiologic - *L. casei*	**Baseline:**<br>800 µg/d FA (n=21)	20.6 ± 5.8[5]	653.6 ± 37.0[6]	287 ± 99[5]
						**Intervention: 4 wks**<br>800 µg/d FA (n=21)	50.2 ±15.6[5]	917.9 ± 49.1[6]	Unavailable
						**Intervention: 8 wks**<br>800 µg/d FA (n=21)	72.2 ± 18.1[5]	1166.2 ± 46.7[6]	226 ± 89[5]
						**Intervention: 12 wks**<br>800 µg/d FA (n=21)	75.4 ± 21.4[5]	1339.4 ± 54.2[6]	Unavailable
						**Intervention: 16 wks**<br>800 µg/d FA (n=21)	77.6 ± 23.0[5]	1430.2 ± 53.1[6]	246 ± 97[5]
**Bronstrup, A. 1998 [13]**	Dietary intervention trial	150 women<br>Age: 20-34[7]	400 µg FA/d, 400 µg FA/d + 6µg vit B-12/d, or 400 µg FA/d + 400 µg vit B-12/d	Plasma folate	Chemiluminescent (Chiron Diagnostics, Fernwald, Germany)	**Baseline:**<br>400 µg FA/d (n=51)<br>400 µg FA/d + 6 µg Vit B-12 (n=49)<br>400 µg FA/d + 400 µg Vit B-12 (n=50)	30.5 ± 10.2[5]<br>31.3 ± 12.9[5]<br>28.8 ± 8.6[5]	Unavailable	Unavailable
						**Intervention: 4wks**<br>400 µg FA/d (n=51)<br>400 µg FA/d + 6 µg Vit B-12 (n=49)<br>400 µg FA/d + 400 µg Vit B-12 (n=50)	46.6 ± 17.4[5]<br>46.1 ± 13.4[5]<br>44.0 ± 11.4[5]	Unavailable	Unavailable
**Brouwer, I. 1998 [14]**	Dietary intervention trial	44 healthy participants<br>Age:18-45[7]	low folate diet plus 250 µg FA/d for 4 wks	Plasma folate	Immunoassay (Abbott IMX)	**Baseline:**<br>250 µg FA/d (n=22)	14.6 ± 4.7[5]	Unavailable	Unavailable
						**Intervention: 4wks**<br>250 µg FA/d (n=22)	20.4 ± 4.1[5]	Unavailable	Unavailable

**Brouwer, I. 1999 [15]**	Randomized controlled trial	144 healthy women<br>Age:18-40[7]<br>Netherlands	250 µg FA/d or 500 µg FA/d for 4wks	Plasma folate	Immunoassay (Abbott IMX)	**Baseline:**<br>250 µg FA (n=45)<br>500 µg FA (n=50)	11 ± 3.4[5]<br>11 ± 4.0[5]	Unavailable	Unavailable
						**Intervention: 4 wks**<br>250 µg FA (n=45)<br>500 µg FA (n=50)	23 ± 5.3[5]<br>27 ± 5.1[5]	Unavailable	Unavailable
**Brouwer, I. 2000 [16]**	Dietary intervention trial	22 women (18-40y)<br>Nijmegen, Netherlands	500 µg FA/2d or 250 µg FA/d for 4 wks	Plasma folate	Immunoassay (Abbott IMX)	**Baseline:**<br>500 µg FA/2d (n=11)<br>250 µg FA/d (n=11)	Unavailable	Unavailable	Unavailable
						**Intervention: 4wks**<br>500 µg FA/2d (n=11)<br>250 µg FA/d (n=11)	9.1 (1.9, 16.3)[12]<br>11.4 (6.8, 15.9)[12]	Unavailable	Unavailable
**Carlsson, CM. 2004 [17]**	Prospective, single-blinded study	27 males and females<br>Age: 70-88[7]	After 10wk placebo all treated with 400 µg FA/d for 10 wks, then a placebo washout for 10 wks, then 1400 µg FA/d for 10 weeks	Serum folate	Chemiluminescent enzymatic immunoassay	**Baseline**<br>400 µg/d (n=20)<br>1400 µg/d (n=17)	24.1 ± 1.4[6,9]<br>27.0 ± 1.6[6,9]	Unavailable	Unavailable
						**Intervention: 10 weeks**<br>400 µg/d (n=20)<br>1400 µg/d (n=17)	37.7 ± 1.6[6,9]<br>43.1 ± 1.4[6,9]	Unavailable	Unavailable
**Cheong, M. 2016 [18]**	Double-blind, placebo-controlled trial	70 non-pregnant women<br>Age: 21-35[7]<br>Singapore	fortified milk powder with 400 µg FA/d for 12 weeks or placebo	Plasma and RBC folate	Microbiologic - *L. casei*	**Baseline:**<br>400 µg FA/d (n=29)	28 ± 14[5]	614 ± 186[5]	Unavailable
						**Intervention: 6 wks**<br>400 µg FA/d (n=29)	71 ± 16[5]	927 ± 242[5]	Unavailable
						**Intervention: 12 wks**<br>400 µg FA/d (n=29)	82 ± 32[5]	1200 ± 311[5]	Unavailable
**Cotlarciuc, I. 2011 [19]**	Dietary intervention trial	101 healthy, female twins<br>Age: 50-80[7]<br>United Kingdom	0.8 mg FA/d for 6 wks	Plasma folate	Chemiluminescent, competitive immunoassay (Bayer Advia Centaur)	**Baseline:**<br>MZ twins (n=51)<br>DZ twins (n=50)	17.3 ± 6.6[5,9]<br>23.6 ± 11.7[5,9]	Unavailable	Unavailable
						**Intervention: 6 weeks**<br>MZ twins (n=51)<br>DZ twins (n=41)	46.5 ± 10.7[5,9]<br>49.3 ± 8.9[5,9]	Unavailable	Unavailable
**Cuskelly, GJ. 1996 [20]**	Randomized, placebo-controlled trial	62 non-pregnant women<br>Age: 17-40[7]<br>United Kingdom	400 µg FA/d for 3 mo	RBC folate	Microbiologic - *L. casei*	**Baseline:**<br>Supplement: 400 µg FA/d (n=9)<br>Fortified food: 400 µg FA/d (n=6)	Unavailable	796.8 ± 136.2[5,9]<br>740.0 ± 104.4[5,9]	209 ± 37[5]<br>186 ± 35[5]
						**Intervention: 3mo**<br>Supplement: 400 µg FA/d (n=9)<br>Fortified food: 400 µg FA/d (n=6)	Unavailable	1116.8 ± 267.9[5,9]<br>1130.5 ± 306.5[5,9]	601 ± 43[5]<br>407 ± 76[5]

**Daly, S. 2002 [21]**	Double-blind, randomized placebo-controlled trial	Women of childbearing age (n=95)<br>Dublin, Ireland	0, 100, 200 or 400 µg FA/d for 10 wks	RBC	Microbiologic - *L. casei*	**Baseline:**<br>100 µg FA/d (n=22)	Unavailable	754.5 ± 199.0[5,9]	Unavailable
						200 µg FA/d (n=28)		716.6 ± 160.9[5,9]	
						400 µg FA/d (n=26)		812.0 ± 243.2[5,9]	
						**Intervention: 10 wks**<br>100 µg FA/d (n=20)	Unavailable	71.2 (-168.2, 205.4)[9,13]	Unavailable
						200 µg FA/d (n=26)		263.3 (57.0, 452.6)[9,13]	
						400 µg FA/d (n=25)		273.0 (106.0, 545.7)[9,13]	
**Davis, BA. 1995 [22]**	Placebo-controlled trial	20 non-pregnant women<br>Age: 12-36[7]<br>Northwestern South Carolina, USA	2000 µg FA/d for 4 wks	Serum and RBC folate	Microbiologic - *L. casei*	**Basline:**<br>2000 µg FA/d (n=10)	7.8 ± 5.6[5]	469 ± 105[5]	Unavailable
						**Intervention: 4 wks**<br>2000 µg FA/d (n=10)	29.4 ± 8.8[5]	549 ± 144[5]	Unavailable
**Deshmukh, U. 2010 [23]**	Cluster randomized, placebo-controlled, double-blind, 2x3 factorial trial	119 families; 294 individuals (106 children, 92 fathers, 96 mothers)<br><br>Age:<br>Children: 9.0 ± 0.2[5]<br>Fathers: 36.8 ± 3.7[5]<br>Mothers: 30.4 ±3.1[5]<br><br>6 rural villages near Pune City, India	0 µg of vitamin B12 and 200 µg FA/d, 2 µg of vitamin B12 and 200 µg FA/d, or 10 µg of vitamin B12 and 200 µg of FA/d	Plasma folate	Microbiologic - *L. casei*	**Baseline:**<br>200 µg FA/d (n=151)	13.5 ± 5.6[5]	Unavailable	Unavailable
						**Intervention: 4 mo**<br>200 µg FA/d (n=151)	33.1 ± 16.1[5]	Unavailable	Unavailable
						**Intervention: 12 mo**<br>200 µg FA/d (n=151)	27.8 ± 14.4[5]	Unavailable	Unavailable
**Dierkes, J. 1998 [24]**	Randomized trial	36 healthy young women<br>Age 24.7± 3.2[5]<br>Germany	400 µg FA/d for 4 wks	Plasma folate	Protein binding test kit, Magic Lite (Ciba Corning Diagnostics GmbH, Fernwald, Germany)	**Baseline:**<br>400 µg FA/d (n=36)	17.8 (5.9, 31.5)[14]	Unavailable	Unavailable
						**Intervention: 4 wks**<br>400 µg FA/d (n=36)	32.9 (15.2, 67.0)[14]	Unavailable	Unavailable

**Doshi, S. 2003 [25]**	Double-blind, randomized, cross-over, controlled trial	50 subjects with CHD<br>Age: 57± 8[5]<br>United Kingdom	5000 µg FA/d for 6 wks	Plasma folate	competing protein binding assay kit	**Baseline:**<br>5000 µg FA/d (n=50)	20.2 ± 7.9[5,9]	Unavailable	Unavailable
						**Intervention: 6 wks**<br>5000 µg FA/d (n=50)	703.7 ± 533.5[5,9]	Unavailable	Unavailable
**Doshi, SN. 2002 [26]**	Randomized, placebo-controlled trial	33 patients<br>Age: 55± 7[5]<br>United Kingdom	5000 µg FA/d for 6 wks	Plasma folate	Competitive binding assay (Abbott IMX)	**Baseline:**<br>5000 µg FA/d (n=16)	22.37 ± 8.7[5]	Unavailable	Unavailable
						**Intervention: 6 wks**<br>5000 µg FA/d (n=16)	604.22 ± 429[5]	Unavailable	Unavailable
**Dragani 2010 [27]**	Dietary intervention trial	41 subjects<br>Age: 42±12[5]<br>Italy	5000 µg FA/d for 8 wks	Serum folate	Radioimmunoassay (ICN, New York, USA)	**Baseline:**<br>5000 µg FA/d (n=23 hyperhomocysteinaemic T allele carriers)	10.0 ± 5.7[5]	Unavailable	Unavailable
						5000 µg FA/d (n=18 MTHFR CC controls)	15.0 ± 7.9[5]		
						**Intervention: 8 wks**<br>5000 µg FA/d (n=23 hyperhomocysteinaemic T allele carriers)	45.5 ± 31.6[5]	Unavailable	Unavailable
						5000 µg FA/d (n=18 MTHFR CC controls)	41.1 ± 18.8[5]		
**Durga, J. 2007 [28]**	Randomized controlled trial	728 men and women<br>Age: 50-70[7]<br>Netherlands	800 µg FA/d for 3 years	RBC and Serum folate	Chemiluminescent immunoassay (Immulite 2000; Diagnostic Products Company, California, USA)	**Baseline:**<br>800 µg/d FA (n=355)	11.5 (9.4, 14.6)[8]	n/a[10]	195 (159, 241)[8]
						**Intervention: 1 year**<br>800 µg/d FA (n=347)	52.7 (43.2, 85.8)[8]	n/a[10]	Unavailable
						**Intervention: 2 years**<br>800 µg/d FA (n=340)	49.4 (41.7, 81.5)[8]	n/a[10]	Unavailable
						**Intervention: 3 years**<br>800 µg/d FA (n=344)	75.0 (50.0, 102.8)[8]	n/a[10]	181 (152, 219)[8]
**Duthie, S. 2010 [29]**	Double-blind, randomized controlled trial	20 participants<br>Age: 39.9± 3.5[5]	1200 µg FA/d for 12 wks	Plasma folate	Chemiluminescence immunoassay (Abbott IMX)	**Baseline:**<br>1200 µg FA (n=10)	15.11 ± 1.58[6]	Unavailable	Unavailable
						**Intervention: 12wks**<br>1200 µg FA (n=10)	92.49 ± 11.71[6]	Unavailable	Unavailable
**Earnest, C. 2002 [30]**	Dietary intervention trial	150 men and women<br>Age: 24-79[7]<br>Texas, USA	800 µg FA/d for 24 weeks	Plasma folate	Microbiologic - *L. casei*	**Baseline:**<br>800 µg FA/d (n=141)	21.07 ± 7.7[5]	Unavailable	Unavailable
						**Intervention: 12 wks**<br>800 µg FA/d (n=141)	29.68 ± 8.6[5]	Unavailable	Unavailable
						**Intervention: 24 wks**<br>800 µg FA/d (n=141)	28.10 ± 9.1[5]	Unavailable	Unavailable
**Ebisch, IMW. 2006 [31]**	Randomized, controlled trial	87 males<br>Age: 30-40[7]<br>Netherlands	5000 µg FA/d and 6600 µg zinc sulphate for 26 wks	Serum folate	Radioassay (Dualcount Solid Phase Boil radioassay, Diagnostic Products Corp., California, USA)	**Baseline:**<br>5000 µg FA/d +zinc (n=18, subfertile male)	18.0 (13.0, 22.8)[8]	Unavailable	Unavailable
						5000 µg FA/d +zinc (n=24, fertile male)	18.0 (15.0, 21.0)[8]		

						**Intervention: 26 wks**			
						5000 µg FA/d +zinc (n=18, subfertile male)	105.0 (86.5, 122.5)[8]	Unavailable	Unavailable
						5000 µg FA/d +zinc (n=24, fertile male)	110.0 (77.0, 130.0)[8]		
**Fohr, I. 2002 [32]**	Randomized control trial	160 healthy, nonpregnant females<br>Age: 19-39[7]<br>Germany	400 µg FA/d for 8 wks	RBC and Plasma folate	Immunoassay (Abbott IMX) for Plasma folate; Microbiologic - *L. casei* for RBC folate	**Baseline:**<br>FA group: 400 µg FA/d (n=51)	15.0 ± 4.9 (14.3)[15]	372 ± 132 (354)[15]	Unavailable
						**Intervention: 4 wks**<br>FA group: 400 µg FA/d (n=51)	24.3 ± 5.8 (23.5)[15]	Unavailable	Unavailable
						**Intervention: 8 wks**<br>FA group: 400 µg FA/d (n=51)	27.9 ± 8.0 (26.6)[15]	608 ± 112 (598)[15]	Unavailable
**Gamble, M. 2006 [33]**	Randomized control trial	194 males, females<br>Age: 18-65[7]<br>Rural region in Bangladesh	400 µg FA/d for 12 wks	Plasma folate	Radioimmunoassay (Quantaphase II; Bio-Rad Laboratories, California, USA)	**Baseline:**<br>Folate group: 400 µg FA/d (n=96)	8.28 ± 4.74[5]	Unavailable	Unavailable
						**Intervention: 12 wks**<br>Folate group: 400 µg FA/d (n=96)	61.57 ± 27.67[5]	Unavailable	Unavailable
**Green, T. 2004 [34]**	Randomized control trial	73 females<br>Age: 18-45[7]<br>Dunedin, New Zealand	400 µg FA/d through fortified milk for 6 wks	RBC and Plasma folate	Microbiologic - *L. casei*	**Baseline:**<br>375 µg FA Fortified milk/d (n=36)	21 (18, 23)[16]	834 (754, 913)[16]	Unavailable
						**Intervention: 6 wks**<br>375 µg FA Fortified milk/d (n=36)	48 (42, 53)[16]	1023 (955, 1091)[16]	889 (862, 915)[16]
						**Intervention: 12 wks**<br>375 µg FA Fortified milk/d (n=36)	51 (46, 56)[16]	1245 (1154, 1335)[16]	Unavailable
**Green, T. 2013 [35]**	Double-blind, randomized controlled trial	30 healthy men/women<br>Age: 18-45[7]<br>Vancouver, Canada	Daily consumption of wheat roll that contains 400µg folic acid for 16 wks	Plasma folate and whole blood folate	Microbiologic - *L. casei*	**Baseline:**<br>400 µg FA (n=14)	40 ± 12[5]	780 ± 190[5]	Unavailable
						**Intervention: 8wks**<br>400 µg FA (n=14)	56 ± 17[5]	1180 ± 310[5]	Unavailable
						**Intervention: 16 wks**<br>400 µg FA (n=14)	59 ± 19[5]	1160 ± 390[5]	Unavailable
**Green, TJ. 2003 [36]**	Dietary intervention trial	78 Women<br>Age:18-49[7]<br>New Zealand	400 µg/d FA for 12 weeks or placebo	RBC folate	Microbiologic - *L. casei*	**Baseline:**<br>400 µg/d FA (n=35)	Unavailable	650 ± 216[5]	224 ± 71[5]
						**Intervention: 12 weeks**<br>400 µg/d FA (n=35)	Unavailable	1104 ± 305[5]	Unavailable
**Grieger, J. 2009 [37]**	Dietary intervention trial	107 elderly participants<br>Age: 79.9± 10.1[5]<br>Victoria, Australia	Daily consumption of fortified milk for 6mo - mean of 120 µg FA/d	Serum folate	Radioimmunoassay (BioRad Laboratories, New South Wales, Australia)	**Baseline:**<br>120 µg FA/d (n=107)	13.4 ± 19.7[5]	Unavailable	Unavailable
						**Intervention: 24 wks**<br>120 µg FA/d (n=107)	17.9 ± 18.1[5]	Unavailable	Unavailable

**Haglund, O. 1993 [38]**	Double-blind, cross-over, dietary intervention trial	12 healthy men<br>Mean age: 48	10 mg FA/d for 4 wks	Serum folate	Radioassay (SimulTRACK, Becton Dickinson, New York, USA)	**Baseline:**<br>10000 µg FA/d (n=12)	16.5 ± 10.1[5]	Unavailable	Unavailable
						**Intervention: 4wks**<br>10000 µg FA/d (n=12)	53.3 ± 2.3[5]	Unavailable	Unavailable
**Hao, L. 2008 [39]**	Randomized control trial	1108 females<br>Age: 24-42[7]<br>Hebei Province, China	100 µg FA/d,<br>400 µg FA/d,<br>4000 µg FA/d,<br>or 4000 µg FA/wk for 6 mo	RBC and Plasma folate	Microbiologic - *L. casei*	**Baseline:**<br>100 µg FA/d (n=339)	9.7 (9.2, 10.2)[17]	594.7 (572.4, 617.8)[17]	Unavailable
						400 µg FA/d (n=338)	9.6 (9.1, 10.2)[17]	603.1 (580.5, 626.6)[17]	
						4000 µg FA/d (n=167)	9.7 (9.0, 10.5)[17]	599.4 (567.7, 632.9)[17]	
						4000 µg FA/wk (n=157)	9.8 (9.0, 10.6)[17]	610.8 (577.5, 646.1)[17]	
						**Intervention: 1 mo**<br>100 µg FA/d (n=339)	18.0 (17.0, 19.0)[17]	630.7 (607.1, 655.3)[17]	Unavailable
						400 µg FA/d (n=338)	29.8 (28.3, 31.5)[17]	728.7 (701.4, 757.1)[17]	
						4000 µg FA/d (n=167)	55.1 (51.0, 59.6)[17]	866.1 (820.2, 914.5)[17]	
						4000 µg FA/wk (n=157)	20.9 (19.3, 22.7)[17]	638.4 (603.6, 675.2)[17]	
						**Intervention: 3 mo**<br>100 µg FA/d (n=339)	19.0 (18.0, 20.1)[17]	659.2 (634.5, 684.8)[17]	Unavailable
						400 µg FA/d (n=338)	36.5 (34.5, 38.5)[17]	914.7 (879.8, 949.7)[17]	
						4000 µg FA/d (n=167)	68.9 (63.8, 74.5)[17]	1342.2 (1271.2, 1417.2)[17]	
						4000 µg FA/wk (n=157)	23.9 (22.0, 25.9)[17]	781.8 (739.2, 827.0)[17]	
						**Intervention: 6 mo**<br>100 µg FA/d (n=339)	20.2 (19.1, 21.3)[17]	759.5 (731.1, 789.1)[17]	Unavailable
						400 µg FA/d (n=338)	34.5 (32.7, 36.5)[17]	1035.5 (996.6,	

								1075.8)[17]	
						4000 µg FA/d (n=167)	54.3 (50.3, 58.7)[17]	1419.8 (1344.6, 1499.1)[17]	
						4000 µg FA/wk (n=157)	23.7 (21.8, 25.6)[17]	888.6 (840.1, 939.9)[17]	
**Herrmann, M. 2006 [40]**	Ranomized controlled trial	61 healthy males and females<br>Age: 58.5 ± 8[5]	Daily 0.4, 1 or 5 mg FA for 2 months	Serum folate	Chemiluminescent immunassay (Bayer Healthcare, Ferwald, Germany)	**Baseline:**<br>0.4 mg FA/d (n=18)<br>1 mg FA/d (n=15)<br>5 mg FA/d (n=17)	 12.49 ± 5.45[5,9]<br>11.12 ± 2.95[5,9]<br>14.30 ± 10.44[5,9]	Unavailable	Unavailable
						**Intervention: 4 weeks**<br>0.4 mg FA/d (n=18)<br>1 mg FA/d (n=15)<br>5 mg FA/d (n=17)	 32.47[18,9]<br>29.13[18,9]<br>76.65[18,9]	Unavailable	Unavailable
						**Intervention: 8 weeks**<br>0.4 mg FA/d (n=18)<br>1 mg FA/d (n=15)<br>5 mg FA/d (n=17)	 50.58[18,9]<br>48.93[18,9]<br>45.19[18,9]	Unavailable	Unavailable
**Heseker, H. 1987 [41]**	Dietary intervention trial	6 healthy subjects<br>Age: 26-43[7]<br>Germany	1000 µg FA/d for 17 wks	Plasma folate	Radioassay (Becton Dickinson)	**Baseline:**<br>1000 µg FA/d (n=6)	12.49[9,18]	Unavailable	Unavailable
						**Intervention: 4wks**<br>1000 µg FA/d (n=6)	24.52[9,18]	Unavailable	Unavailable
						**Intervention: 15wks**<br>1000 µg FA/d (n=6)	29.06[9,18]	Unavailable	Unavailable
						**Intervention: 17 wks**<br>1000 µg FA/d (n=6)	29.51[9,18]	Unavailable	Unavailable
**Heseker, H. 1993 [42]**	Double-blind, randomized, placebo-controlled trial	498 men<br>Age: 17-29[7]	800 µg F/d for 8 wks	Plasma folate	Radioassay (Clinical Assays)	**Baseline:**<br>800 µg F/d (n=498)	15.80 (0.24)[6]	Unavailable	Unavailable
						**Intervention: 8 wks**<br>800 µg F/d (n=498)	34.00 (0.62)[6]	Unavailable	Unavailable
**Hiraoka, M. 2004 [43]**	Dietary intervention trial	250 healthy, nonpregnant females<br>Age: 19-29[7]<br>Japan	2 wk baseline period was followed by two 4 wk treatment periods (200 and 400 µg/d FA supplementation) with a 4 wk washout period in between	Serum folate	Chemiluminescence immunoassay (ACS Folate II, ADVIA Centaur, Bayer Medical)	**Baseline:**<br>Young women (n=100)	19.64 ± 10.21[5]	Unavailable	316.37 ± 100.95[5]
						**Intervention: 6 wks**<br>200 µg/d (n=100)	27.51 ± 13.1[5]	Unavailable	318.08 ± 137.71[5]
						**Intervention: 14 wks**<br>400 µg/d (n=100)	34.0 ±8.64[5]	Unavailable	311.94 ± 136.69[5]
**Holmes, T. 2003 [44]**	Randomized controlled trial	41 Caucasian men<br>Age: >58<br>Oklahoma, USA<br>October 1999-June 2000	fortified cereals containing 400 µg per serving per day	Serum folate	Radioimmunoassay (Cobra II, Auto-Gamma Counter; Packard Instrument Co.)	**Baseline:**<br>400 µg FA (n=21)	22.7 ± 6.8[5,9]	Unavailable	Unavailable

						**Intervention: 4wks**<br>400 µg FA (n=21)	29.5 ± 6.8[5,9]	Unavailable	Unavailable
						**Intervention: 8wks**<br>400 µg FA (n=21)	29.5 ± 4.5[5,9]	Unavailable	Unavailable
						**Intervention: 20 wks**<br>400 µg FA (n=21)	34.1 ± 6.8[5,9]	Unavailable	Unavailable
**Hursthouse, N. 2011 [45]**	Randomized control trial	119 healthy females<br>Age: 18-40[7]<br>New Zealand<br>Aµgust 2008 - May 2009	140 µg FA/d or 400 µg FA/d for 40 wks	RBC and Plasma folate	Microbiologic Assay- *L. rhamnosus* (ATCC 27773; American Type Culture Collection, Manassas, VA)	**Baseline:**<br>140 µg FA/d: (n=49)	19.5 (16.5, 22.5)[17]	711.8 (626.1, 797.5)[17]	248.0 ± 1.6[19]
						400 µg FA/d: (n=48)	19.2 (16.3, 22.2)[17]	755.5 (664.2, 846.8)[17]	271.5 ± 1.5[19]
						**Intervention: 6 wks**<br>140 µg FA/d: (n=47)	26.6 (22.5, 30.8)[17]	834.5 (732.8, 936.1)[17]	Unavailable
						400 µg FA/d: (n=42)	37.3 (31.2, 43.3)[17]	910.8 (796.2, 1025.3)[17]	
						**Intervention: 12 wks**<br>140 µg FA/d: (n=44)	28.0 (23.6, 32.5)[17]	849.0 (743.4, 954.7)[17]	Unavailable
						400 µg FA/d: (n=41)	38.3 (32.1, 44.6)[17]	940.1 (821.2, 1059.1)[17]	
						**Intervention: 29 wks**<br>140 µg FA/d: (n=41)	31.0 (25.9, 36.0)[17]	987.0 (861.8, 1112.2)[17]	Unavailable
						400 µg FA/d: (n=40)	37.3 (31.1, 43.4)[17]	1121.7 (977.9, 1265.4)[17]	
						**Intervention: 40 wks**<br>140 µg FA/d: (n=42)	29.0 (24.3, 33.7)[17]	1111.3 (970.3, 1252.3)[17]	Unavailable
						400 µg FA/d: (n=39)	39.5 (33.0, 46.1)[17]	1273.4 (1110.2, 1436.6)[17]	
**Johansson, M. 2002 [46]**	Dietary intervention trial	29 healthy women<br>Age: 23-50[7]<br>Sweden<br>January 2001	Wheat breakfast rolls that contained 166 µg FA. One roll eaten per day	Serum folate	Protein binding (Abbott IMX)	**Baseline:**<br>166 µg FA (n=14)	16.9 ± 4.3[5]	Unavailable	Unavailable
						**Intervention: 4 wks**<br>166 µg FA (n=14)	21.2 ± 2.4[5]	Unavailable	Unavailable

						**Intervention: 8 wks**<br>166 µg FA (n=14)	22.4 ± 1.3[5]	Unavailable	Unavailable
						**Intervention: 12 wks**<br>166 µg FA (n=14)	22.0 ± 2.1[5]	Unavailable	Unavailable
**Kauwell, G. 2000 [47]**	Dietary intervention trial	33 healthy women<br>Age: 60-85[7]<br>Florida, USA	7 weeks of 82 or 301 µg FA/d throµgh folate restricted diet and FA throµgh apple juice.	Serum folate	Microbiologic - *L. casei*	**Baseline:**<br>82 µg FA (n=8)<br>301 µg FA (n=7)	10.8 ± 4.4[5]<br>15.8 ± 6.9[5]	Unavailable	Unavailable
						**Intervention: 7 wks**<br>82 µg FA (n=7)<br>301 µg FA (n=8)	16.2 ± 10.0[5]<br>32.0 ± 10.6[5]	Unavailable	Unavailable
**Klerk, M. 2004 [48]**	Randomized control trial	276 males and females<br>Age: 50-70[7]<br>Sept - Dec 2000	0.8 mg FA/d for 1 year	Serum folate	Chemiluminescent immunoassay analyzer (Immulite 2000; Diagnostic Products Company, Los Angeles)	**Baseline:**<br>0.8 mg/d FA (n=137)	12.4 (10.2, 15.6)[8]	Unavailable	Unavailable
						**Intervention: 1 yr**<br>0.8 mg/d FA (n=137)	49.5 (42.3, 90.3)[8]	Unavailable	Unavailable
**Kok, DEG. 2015 [49]**	Double-blind, randomized, placbo-controlled trial	92 participants<br>Age: 65-75[7]<br>Netherlands	400 µg FA/d or placebo for 2 years	Serum folate	Electro-chemiluminescence immunoassay (Elecsys 2010, Roche, Almere, Netherlands)	**Baseline:**<br>400 µg FA/d (n=44)	16.2 (13.1, 24.4)[8]	Unavailable	Unavailable
						**Intervention: 2 years**<br>400 µg FA/d (n=44)	52.3 (45.1, 67.6)[8]	Unavailable	Unavailable
**Lamers, Y. 2004 [50]**	Controlled trial	34 female<br>Age: 18 – 25[7]<br>Bonn, Germany<br>Jan - July 2002	**Intervention:** FA and MTHF supplement feeding study (24wk)<br><br>Dietary intake using 3-d diet records at baseline, weeks 8, 16, 24	Plasma folate	Immunoassay (AxSYM Analyzer, Abbott Laboratories)	**Baseline**<br>400 µg FA/d (n=34)	14.4 (12.6, 16.5)[17]	Unavailable	Unavailable
						**Intervention: 4 wks**<br>400 µg FA/d (n=34)	28.8 (26.3, 31.5)[17]	Unavailable	Unavailable
						**Intervention: 8 wks**<br>400 µg FA/d (n=34)	34.9 (32.6, 37.4)[17]	Unavailable	Unavailable
						**Intervention: 12 wks**<br>400 µg FA/d (n=34)	35.7 (33.4, 38.0)[17]	Unavailable	Unavailable
						**Intervention: 16 wks**<br>400 µg FA/d (n=34)	37.1 (34.4, 40.0)[17]	Unavailable	Unavailable
						**Intervention: 20 wks**<br>400 µg FA/d (n=34)	37.6 (35.5, 39.9)[17]	Unavailable	Unavailable
						**Intervention: 24 wks**<br>400 µg FA/d (n=34)	34.7 (32.6, 37.0)[17]	Unavailable	Unavailable
**Liem, A. 2003 [51]**	Randomized controlled trial	593 cardiology patients,<br>Age: 64.9± 9.9[5]	500 µg FA/d for 36 months	Serum folate	Ion capture (Abbott IMX)	**Baseline:**<br>500 µg FA/d (n=300)	17 ± 7[5]	Unavailable	Unavailable
						**Intervention: 12 wks**<br>500 µg FA/d (n=300)	33 ± 6[5]	Unavailable	Unavailable
**Lin, P. 2006 [52]**	Randomized, placebo-controlled trial	46 cardiology patients<br>Age: 74.6 ±7.8[5]<br>Taiwan	240 µg FA/d for 8 wks	Serum folate	Chemiluminescent Immunoassay	**Baseline:**<br>240 µg FA/d (n=24)	22.2 ± 10.9[5]	Unavailable	Unavailable

						**Intervention: 4 wks**<br>240 µg FA/d (n=24)	36.3 ± 18.4[5]	Unavailable	Unavailable
						**Intervention: 8 wks**<br>240 µg FA/d (n=24)	41.7 ± 16.8[5]	Unavailable	Unavailable
**Mann, BA. 1987 [53]**	Randomized, double-blind, placebo-controlled trial	101 elderly men/women,<br>Age: 59-81[7]<br>New Mexico, USA	400 µg FA/d for 16 weeks	Plasma folate	Radioassay (Becton Dickinson Immunodiagnostics, New York, USA)	**Baseline:**<br>400 µg FA/d (n=25) men<br>400 µg FA/d (n=28) women	19.1 ± 7.9[5,9]<br>17.3 ± 5.7[5,9]	Unavailable	Unavailable
						**Intervention: 8 wks**<br>400 µg FA/d (n=25) men<br>400 µg FA/d (n=28) women	33.1 ± 13.2[5,9]<br>28.6 ± 7.3[5,9]	Unavailable	Unavailable
						**Intervention: 16 wks**<br>400 µg FA/d (n=25) men<br>400 µg FA/d (n=28) women	32.5 ± 5.4[5,9]<br>37.9 ± 10.0[5,9]	Unavailable	Unavailable
**Mao, G. 2008 [54]**	Double-blind, randomized controlled trial	480 patients with mild-moderate primary hypertension<br>Age: 27-75[7]<br>China	10 mg of enalapril + 400µg FA/d, 10 mg of enalapril + 800 µg FA/d, or placebo for 8wks	Serum folate	Chemiluminescent immunoassay (Beckman-Coulter Canada, Inc., Mississaµga, Canada)	**Baseline:**<br>400 µg FA/d + enalapril (n=146)<br>800 µg FA/d + enalapril (n=148)	12.6 ± 1.5[19]<br>12.5 ± 1.5[19]	Unavailable	Unavailable
						**Intervention: 8wks**<br>400 µg FA/d + enalapril (n=146)<br>800 µg FA/d + enalapril (n=148)	23.7 (1.5)[19]<br>26.7 (1.5)[19]	Unavailable	Unavailable
**Martin-Bautista, E. 2010 [55]**	Double-blind, randomized controlled trial	72 participants<br>Age: 30-65[7]<br>Granada, Spain	150 µg FA/d throµgh enriched milk or control milk for 1 yr	Serum folate	Radioimmunoassay (SimulTrac-SNB radioassay; MP Biomedicals, Ohio, USA)	**Baseline:**<br>150 µg FA/d (n=39)	47.7 ± 4.9[6,9]	Unavailable	Unavailable
						**Intervention: 12 wks**<br>150 µg FA/d (n=39)	66.9 ± 4.4[6,9]	Unavailable	Unavailable
						**Intervention: 24 wks**<br>150 µg FA/d (n=39)	86.5 ± 6.3[6,9]	Unavailable	Unavailable
						**Intervention: 48 wks**<br>150 µg FA/d (n=39)	72.9 ± 4.5[6,9]	Unavailable	Unavailable
**McKay, DL. 2000 [56]**	Randomized, double-blind, placebo-controlled trial	80 free living men and women<br>Age: 50-70[7]<br>Boston, MA, USA	400 µg FA/d for 8 wks or placebo	Plasma folate	Radioimmunoassay (Quantaphase II; BioRad Laboratories, California, USA)	**Baseline:**<br>400 µg FA/d (n=41)	23.3 ± 7.5[5]	Unavailable	378 ± 209[5]
						**Intervention: 8 wks**<br>400 µg FA/d (n=41)	33.0 ± 8.4[5]	Unavailable	Unavailable
**Meer, K. 2005 [57]**	Double-blind, randomized controlled trial	12 healthy volunteers<br>Age: 18-30 years or older than 50 years<br>Amsterdam	400 µg FA/d for 5 wks	Serum folate	Chemiluminescent immunoassay (Chiron Diagnostics, Halstead, UK)	**Baseline:**<br>400 µg FA (<30y, n=6)<br>400 µg FA (>50y, n=6)	13.3 (8.9-23.1)[11]<br>16.7 (6.4-20.7)[11]	Unavailable	Unavailable
						**Intervention: 5wks**<br>400 µg FA (<30y, n=6)<br>400 µg FA (>50y, n=6)	22.1 (10.1-30.0)[11]<br>23.7 (18.3-30.5)[11]	Unavailable	Unavailable

**Melse-Boonstra, A. 2004 [58]**	Randomized control trial	180 males, females<br>Age: 50-75[7]<br>Netherlands	145 µg of hepatoglutamyl FA/d or 320 µg of monoglutamyl FA/d for 12 wks	RBC and Serum folate	Chemiluminescent immunoassay (Immulite 2000; Diagnostic Products Company, Los Angeles)	**Baseline:**			Unavailable
						323 nmol hepatoglutamyl FA/d (145 µg): (n=60)	14.4 ± 5.4[5]	n/a[10]	
						262 nmol monoglutamyl FA/d (320 µg): (n=59)	15.2 ± 4.1[5]		
						**Intervention: Week 2**			Unavailable
						323 nmol hepatoglutamyl FA/d (145 µg): (n=60)	15.8 ± 4.6[5]	n/a[10]	
						262 nmol monoglutamyl FA/d (320 µg): (n=58)	18.8 ± 4.7[5]		
						**Intervention: Week 12**			Unavailable
						323 nmol hepatoglutamyl FA/d (145 µg): (n=60)	19.3 ± 5.6[5]	n/a[10]	
						262 nmol monoglutamyl FA/d (320 µg): (n=59)	25.7 ± 7.4[5]		
**Nguyen, P. 2008 [59]**	Randomized control trial	459 females<br>Age: 15-49[7]<br>Guatemala	12 wks of the following intake:<br>Groups -<br>1: 5000 µg FA, 120 mg Fe, 30 mg Zn, 16.8 µg B12 WEEKLY<br><br>2: 2800 µg FA, 120 mg Fe, 0 mg Zn, 16.8 µg B12 WEEKLY<br><br>3: 400 µg FA, 60 mg Fe, 15 mg Zn, 2.4 µg B12 DAILY<br><br>4: 200 µg FA, 60 mg Fe, 0 mg Zn, 2.4 µg B12 DAILY	Serum folate	Microbiologic - *L. casei*	**Baseline:**			
						Group 1: 5000 µg weekly (n=87)	31.0 (28.4, 33.6)[20]	Unavailable	384 (292-524)[8]
						Group 2: 2800 µg weekly (n=91)	31.8 (29.3, 34.3)[20]		370 (252-485)[8]
						Group 3: 400 µg daily (n=80)	33.1 (30.4, 35.7)[20]		340 (246-540)[8]
						Group 4: 200 µg daily (n=96)	31.1 (28.7, 33.6)[20]		364 (281-483)[8]
						**Intervention: 12 wks**			
						Group 1: 5000 µg weekly (n=86)	46.2 (44.1, 48.4)[20]	Unavailable	Unavailable
						Group 2: 2800 µg weekly (n=90)	46.2 (44.1, 48.3)[20]		
						Group 3: 400 µg daily (n=79)	47.9 (45.7, 50.2)[20]		
						Group 4: 200 µg daily (n=96)	48.1 (46.1, 50.1)[20]		
**Nguyen, P. 2009 [60]**	Dietary intervention trial	40 nonpregnant, healthy females<br>Age: 18-45[7]<br>Toronto, ON, Canada<br>March 2007 - February 2008	1.1 mg FA/d or 5 mg FA/d for 30 wks	RBC and Plasma folate	Microbiological assay with *Lactobacillus rhamnosus* (ATCC 7469; American Type Tissue Culture Collection, Manassas, VA)	**Baseline:**			
						1.1 mg FA/d (n=20)	49.4 ± 15.7[5]	1035 ± 273[5]	376 ± 128[5]
						5 mg FA/d (n=20)	48.8 ± 14.5[5]	1121 ± 410[5]	353 ± 129[5]
						**Intervention: 30 wks**			
						1.1 mg FA/d (n=19)	96.8 ± 41.1[5]	1625 ± 339[5]	357 ± 132[5]
						5 mg FA/d (n=19)	165.3 ± 109.9[5]	2339 ± 782[5]	380 ± 170[5]
**Norsworthy, B. 2001 [61]**	Randomized controlled trial	114 nonpregnant women<br>Age: 18-40[7]<br>Dunedin, New Zealand<br>July 2000- October 2000	2800 µg FA/wk or 400 µg FA/d for 12 wks	Plasma folate and RBC	Microbiologic - *L. casei*	**Baseline:**			
						2800 µg FA/wk (n=37)	20 (17, 24)[17]	608 (553, 668)[17]	Unavailable
						400 µg FA/d (n=35)	18 (16, 21)[17]		

								615 (560, 677)[17]	
						**Intervention: 6 wks**			
						2800 µg FA/wk (n=37)	28 (25, 32)[17]	747 (685, 816)[17]	Unavailable
						400 µg FA/d (n=35)	38 (32, 43)[17]	863 (797, 935)[17]	
						**Intervention: 12 wks**			
						2800 µg FA/wk (n=37)	32 (28, 36)[17]	900 (828, 978)[17]	Unavailable
						400 µg FA/d (n=35)	44 (39, 49)[17]	1053 (957, 1158)[17]	
**Olthof, M. 2006 [62]**	Randomized, double blind, crossover, control trial	40 healthy males and females<br>Age: 50-70[7]<br>Wageningen University in the Netherlands	0.8 mg FA/d for 6 wks	Serum folate	Chemiluminescent immunoassay (IMMULITE 2000)	**Baseline:**			
						0.8 mg FA/d (n=40)	16.8 ± 7.9[5]	Unavailable	Unavailable
						**Intervention: 6 wks**			
						0.8 mg FA/d (n=39)	53.0 ± 17.7[5]	Unavailable	Unavailable
**Oort, F. 2003 [63]**	Double-blind, randomized controlled trial	316 men and women<br>Age: 50-75[7]<br>Wageningen, Netherlands	50, 100, 200, 400, 600, 800 µg FA/d, or placebo for 12 wks	Serum folate	Chemiluminescent immunoassay (Immulite 2000, Diagnostic Products Company)	**Baseline:**			
						50 µg FA (n=42)	12.0 ± 3.1[5]	Unavailable	Unavailable
						100 µg FA (n=41)	12.7 ± 4.6[5]		
						200 µg FA (n=43)	12.3 ± 4.2[5]		
						400 µg FA (n=43)	13.8 ± 5.3[5]		
						600 µg FA (n=43)	12.9 ± 4.9[5]		
						800 µg FA (n=43)	12.9 ± 3.6[5]		
						**Intervention: 4wks**			
						50 µg FA (n=42)	14.7 ± 3.7[5]	Unavailable	Unavailable
						100 µg FA (n=41)	17.4 ± 6.2[5]		
						200 µg FA (n=43)	19.8 ± 5.7[5]		
						400 µg FA (n=43)	31.9 ± 15.3[5]		
						600 µg FA (n=43)	41.4 ± 19.4[5]		
						800 µg FA (n=43)	53.4 ± 28.3[5]		
						**Intervention: 12 wks**			
						50 µg FA (n=42)	16.3 ± 4.4[5]	Unavailable	Unavailable
						100 µg FA (n=41)	19.9 ± 7.6[5]		
						200 µg FA (n=43)	24.6 ± 7.5[5]		
						400 µg FA (n=43)	43.2 ± 21.0[5]		
						600 µg FA (n=43)	55.6 ± 24.5[5]		
						800 µg FA (n=43)	74.8 ± 43.3[5]		
**Papandreou, D. 2010 [64]**	Dietary intervention trial	524 children<br>Age: 6-15[7]<br>8 public schools in Northern Greece	5 mg oral FA 2 times/wk	Serum folate	Immunoassay (Abbott IMX)	**Baseline:**			
						5mg oral FA 2x/wk (n=20)	9.8 (6.8 - 45.4)[9.21]	Unavailable	328 (170-627)[21]
						**Intervention: 2 mo**			
						5mg oral FA 2x/wk (n=20)	38.1 (15.9 - 45.4)[9.21]	Unavailable	388 (270-840)[21]

**Pathansali, R. 2006 [65]**	Randomized control trial	24 healthy, elderly males, females<br>Age: ≥ 65	5 mg FA/d for 4 wks	Serum folate	Competitive protein binding immunoassays (Bayer Diagnostics, Newbury, Berkshire, UK)	**Baseline:**<br>5 mg/d FA: (n=12)	13.62 ± 5.22[5,9]	Unavailable	Unavailable
						**Intervention: 4 wks**<br>5 mg/d FA: (n=12)	45.17 ± 1.36[5,9]	Unavailable	Unavailable
**Pena, A. 2007 [66]**	Double-blind, randomized controlled trial	53 obese adolescents<br>Age: 13.0 ± 2.18[5]<br>Adelaide, Australia<br>January 2004- January 2006	5000 µg FA/d or placebo for 8wks	Serum folate	Chemiluminescent microparticle folate-binding protein assay (Abbott Laboratories)	**Baseline:**<br>5000 µg FA/d (n=27)	18.60 ± 9.72[5]	Unavailable	Unavailable
						**Intervention: 8 wks**<br>5000 µg FA/d (n=27)		Unavailable	Unavailable
**Petrogianni, M. 2012 [67]**	Randomized control trial	101 Hypercholesterolaemic adults<br>Age: 40-60[7]<br>Athens, Greece	500 µg FA/d through consumption of enriched milk for 3 mo	Plasma folate	Electrochemiluminescence (Roche E170)	**Baseline:**<br>500 µg FA/d (n=40)	19.1 ± 7.5 [9,5]	Unavailable	Unavailable
						**Intervention: 3 mo**<br>500 µg FA/d (n=40)	32.9 ± 9.5 [9,5]	Unavailable	Unavailable
**Pinto, X. 2005 [68]**	Randomized, crossover, dietary intervention trial	20 male patients with history of coronary artery disease<br>Age: 60.9± 8.91[5]<br>Spain	500 µg FA/d for 5 wks or 370 µg food folate & 130 µg FA; followed by 5 wks washout then switch	Serum folate	Radioassay (SimulTRACK, Becton Dickinson, New York, USA)	**Baseline:**<br>500 µg FA/d (n=20)<br>370 µg food folate + 130 µg FA/d (n=20)	19.42 ± 7.53[5]<br>18.89 ± 6.46[5]	Unavailable	Unavailable
						**Intervention: 5 wks**<br>500 µg FA/d (n=20)<br>370 µg food folate + 130 µg FA/d (n=20)	34.53 ± 10.12[5]<br>27.55 ± 9.48[5]	Unavailable	Unavailable
**Pullin, CH. 2001 [69]**	Crossover, dietary intervention trial	Healthy subjects (n=126), 42 of each MTHFR genotype<br>Age: 18-65[7]<br>South Wales, United Kingdom	400 µg FA/d for 4 mo	Plasma folate	Abbott IMX methods	**Baseline:**<br>CC (n=42)<br>CT (n=42)<br>TT (n=42)	19.1 ± 7.0[5,9]<br>17.7 ± 6.8[5,9]<br>14.8 ± 7.3[5,9]	Unavailable	254 ± 83[5]
						**Intervention: 4 mo**<br>CC (n=35)<br>CT (n=35)<br>TT (n=36)	36.8 ± 25.2[5,9]<br>30.2 ± 8.4[5,9]<br>27.9 ± 8.6[5,9]	Unavailable	561 ± 98[5]
**Qin, X. 2012 [70]**	Randomized control trial	480 Chinese adults with mild or moderate hypertension<br>Age: 18-75[7]<br>Six hospitals in different Chinese regions (Ha'erbin, Shanghai, Shenyang, Beijing, Xi'an, and Nanjing)<br>Sept - Dec 2005	Once per day Enalapril supplement with<br>a) no FA<br>b) with 0.4 mg FA<br>c) with 0.8 mg FA<br>(8 wks)	Serum folate	Chemiluminescent immunoassay (Beckman-Coulter Canada Inc., Mississaμga, Canada)	**Baseline:**<br>Low-FA: Enalapril 10 mg combined with 0.4 mg FA /d (n=147)<br>High-FA: Enalapril 10 mg combined with 0.8 mg FA /d (n=145)	13.7 ± 6.1[5]<br>13.5 ± 6.3[5]	Unavailable	Unavailable
						**Intervention: 4 wks**<br>Low-FA: Enalapril 10 mg combined with 0.4 mg FA /d (n=147)<br>High-FA: Enalapril 10 mg combined with 0.8 mg FA /d (n=145)	21.4 ± 9.2[5]<br>25.9 ± 11.0[5]	Unavailable	Unavailable

						**Intervention: 8 wks**			
						Low-FA: Enalapril 10 mg combined with 0.4 mg FA /d (n=147)	24.1 ± 10.6[5]	Unavailable	Unavailable
						High-FA: Enalapril 10 mg combined with 0.8 mg FA /d (n=145)	26.9 ± 10.9[5]		
**Quinlivan, EP. 2002 [71]**	Dietary intervention trial	30 healthy men with normal concentrations of folate and vitamin B12<br>Age: 34-65[7]	100 µg FA/d for 6 wks	Plasma folate	Microbiologic- *L. casei*	**Baseline:**<br>Preintervention (n=30)	14.3 (11.4, 22.7)[8,9]	Unavailable	Unavailable
						**Intervention: 6 wks**<br>100 µg FA/d (n=30)	18.8 (15.0, 25.4)[8,9]	Unavailable	Unavailable
						**Intervention: 12 wks**<br>200 µg FA/d (n=30)	24.4 (19.7, 33.1)[8,9]	Unavailable	Unavailable
						**Intervention: 26 wks**<br>400 µg FA/d (n=30)	40.1 (29.7, 46.1)[8,9]	Unavailable	Unavailable
		23 healthy women with normal concentrations of folate and vit B12	500 µg FA/d for 4 mo			**Baseline:**<br>500 µg FA/d (n=23)	19.5 (10.0, 23.8)[8,9]	Unavailable	Unavailable
						**Intervention: 4 mo**<br>500 µg FA/d (n=23)	35.2 (21.1, 40.4)[8,9]	Unavailable	Unavailable
**Raigani, M. 2014 [72]**	Randomized, placebo-controlled trial	83 infertile men<br>Iran	5000 µg FA and placebo, FA and zinc sulphate, or placebo and placebo for 16 wks	Serum folate	radioimmunoassay (MP Biomedicals Simul-TRAC-SNB, New York USA)	**Baseline:**<br>5000 µg FA/placebo (n=20)<br>5000 µg FA/Zinc sulphate (n=21)	9.1 (5.9, 17.3)[8,9]<br>9.5 (7.0, 14.8)[8,9]	Unavailable	Unavailable
						**Intervention: 16 wks**<br>5000 µg FA/placebo (n=20)<br>5000 µg FA/Zinc sulphate (n=21)	73.5 (33.8, 110.5)[8,9]<br>76.0 (27.3, 154.4)[8,9]	Unavailable	Unavailable
**Rhode, BM. 1983 [73]**	Dietary intervention trial	57 non pregnant women	60 µg FA/d for 7 wks	Serum folate	Microbiologic - *L. casei*	**Baseline:**<br>60 µg FA/d (n=20)	19.1 ± 1.6[6,9]	Unavailable	Unavailable
						**Intervention: 7 wks**<br>60 µg FA/d (n=20)	46.5 ± 13.2[6,9]	Unavailable	Unavailable
**Rosenthal, J. 2008 [74]**	Randomized, double-blind control trial	140 women<br>Age:18-49[7]<br>Choloma, Honduras	1 mg FA/d or 5mg FA/wk for 12 weeks	Serum folate and RBC folate	Radioassay	**Baseline:**<br>5 mg/week (n=58)<br>1 mg/d (n=50)	15.66 ± 0.68[6,9]<br>14.30 ± 0.45[6,9]	n/a[10]	Unavailable
						**Intervention: 6 wks**<br>5 mg/week (n=58)<br>1 mg/d (n=50)	19.75 ± 0.68[6,9]<br>27.69 ± 0.68[6,9]	n/a[10]	Unavailable
						**Intervention: 12 wks**<br>5 mg/week (n=58)<br>1 mg/d (n=50)	22.93 ± 0.91[6,9]<br>33.82 ± 1.36[6,9]	n/a[10]	Unavailable
**Schorah, CJ. 1998 [75]**	Randomized, double-blind, placebo-controlled trial	119 healthy volunteers aged 32-65 years<br>Leeds, England	200 µg FA/d for 24 wks	Serum and RBC folate	Radioassay (Diagnostic Products Corporation)	**Baseline:**<br>200 µg FA/d (n=33)<br>200 µg FA/d + other vitamins (n=31)	19.7 (13.8, 23.2)[9,22]<br>17.5 (14.3, 20.7)[9,22]	n/a[10]	Unavailable
						**Intervention: 4 wks**<br>200 µg FA/d (n=33)<br>200 µg FA/d + other vitamins (n=31)	29.1 (22.2, 34.7)[9,22]<br>23.6 (18.4, 27.9)[22]	n/a[10]	Unavailable
						**Intervention: 8 wks**<br>200 µg FA/d (n=33)<br>200 µg FA/d + other vitamins (n=31)	26.1 (18.8, 25.2)[9,22]<br>24.1 (17.5, 28.6)[9,22]	n/a[10]	Unavailable

						**Intervention: 24 wks**<br>200 µg FA/d (n=33)<br>200 µg FA/d + other vitamins (n=31)	 31.3 (20.9, 40.0)[9,22]<br>31.1 (20.7, 40.6)[9,22]	 n/a[10]	 Unavailable
**Sharifi, F. 2010 [76]**	Randomized, double-blind, placebo-controlled trial	42 hypertensive individuals, Age: 25-64[7]<br>Tehran, Iran<br>2006	5000 µg FA/d for 6 weeks	Serum folate	Radioimmunoassay (SimulTrac, ICN Pharmaceuticals)	**Baseline:**<br>5000 µg FA/d (n=18)	 16.1 ± 7.0[5,9]	 Unavailable	 Unavailable
						**Intervention: 6 wks**<br>5000 µg FA/d (n=18)	 57.4 ± 7.0[5,9]	 Unavailable	 Unavailable
**Sheu, W. 2005 [77]**	Randomized control trial	74 obese females with BMI 29.6 ± 0.5 kgs/m2<br>Age: 41 ± 1[6]<br>Taiwan	5 mg FA/d for 12 wks	Serum folate	Chemiluminescence kits within one run (IMMULITE, Diagnostic Products Corporation, Los Angeles, CA, USA)	**Baseline:**<br>5 mg FA/d: (n=36)	 22.2 ± 1.5[6]	 Unavailable	 Unavailable
						**Intervention: wk 12**<br>5 mg FA/d: (n=36)	 69.8 ± 10.1[6]	 Unavailable	 Unavailable
**Shidfar, F. 2009 [78]**	Randomized control trial	40 asymptomatic newly diagnosed hypercholesterolemic males and females<br>Mean age: 44.87 ± 7.33[5]<br>Tehran, Iran	5 mg FA/d for 8 wks	Plasma folate	Radioimmunoassay	**Baseline:**<br>5 mg/d (n=20)	 5.08 ± 2.01[5]	 Unavailable	 Unavailable
						**Intervention: 8 wks**<br>5 mg/d (n=20)	 14.29 ± 5.94[5]	 Unavailable	 Unavailable
**Smidt, LJ. 1990 [79]**	Double-blind, randomized, placebo-controlled trial	80 women<br>Age: 65-92[7]<br>Cork City, Ireland	400 µg FA/d for 6 wks	Plasma folate	Microbiologic - *L. casei*	**Baseline:**<br>400 µg FA/d (n=20)<br>400 µg FA/d + 10 µg thiamin (n=20)	 13.6 ± 8.2[5]<br>9.1 ± 6.3[5]	 Unavailable	 Unavailable
						**Intervention: 6wks**<br>400 µg FA/d (n=20)<br>400 µg FA/d +10 µg thiamin (n=20)	 43.0 ± 22.2[5]<br>40.8 ± 18.8[5]	 Unavailable	 Unavailable
**Strandhagen, E. 2003 [80]**	Randomized control Trial	121 healthy, nonsmoking males and females<br>Age: 29-65[7]<br>Gothenburg, Sweden	200 µg FA/d for 4 wks	Serum folate	Immunoassay (ADVIA Centaur)	**Baseline:**<br>200 µg FA/d (n=59)	 11.9 ± 6.5[5]	 Unavailable	 Unavailable
						**Intervention: week 4**<br>200 µg FA/d (n=59)	 20.8 ± 6.07[5]	 Unavailable	 Unavailable
**Sweeney, M.R. 2007 [81]**	Dietary intervention trial	20 healthy adult subjects (10 male, 10 female)<br>Age: 20-40[7]<br>Dublin, Ireland	400 µg FA/d for 14 wks	RBC and Serum folate	Microbiologic- *L. casei*	**Baseline:**<br>400 µg FA/d (n=19)	 17.25 ± 9.65[5,9]	 1084.99 ± 394.96[5,9]	 Unavailable
						**Intervention: 14 weeks**<br>400 µg FA/d (n=19)	 33.53 ± 11.37[5,9]	 1575.24 ± 451.09[5,9]	 Unavailable
**Tapola, NS. 2004 [82]**	Randomized, controlled, double-blinded, parallel trial	n=60 men and women<br>Age: 48± 11[5]<br>Eastern Finland	Mineral water fortified with 563 µg/d FA for 8 weeks or control	RBC and Serum folate	AutoDELFIA Folate time-resolved fluoroimmunoassay method (TR-FIA)	**Baseline:**<br>563 µg/d FA (n=31)	 12.1 ± 4.4[5]	 n/a[10]	 324 ± 91[5]
						**Intervention: 2 wks**<br>563 µg/d FA (n=31)	 20.0 ± 6.9[5]	 n/a[10]	Unavailable

						**Intervention: 4 wks**<br>563 µg/d FA (n=31)	24.5 ± 7.6[5]	n/a[10]	875 ± 99[5]
						**Intervention: 8 wks**<br>563 µg/d FA (n=31)	28.2 ± 7.3[5]	n/a[10]	Unavailable
**Tighe, T. 2011 [83]**	Double-blind, randomized, placebo-controlled trial	172 patients<br>Northern Ireland	placebo, 0.2, 0.4, 0.8 mg FA/day for 26 wks	Serum folate	Microbiologic - *L. casei*	**Baseline:**<br>2000 µg FA (n=44)<br>4000 µg FA (n=43)<br>8000 µg FA (n=45)	14.8 (14.1, 20.4)[9,17]<br>17.7 (16.6, 22.9)[9,17]<br>17.3 (16.6, 22.9)[9,17]	Unavailable	Unavailable
						**Intervention: 26 wks**<br>2000 µg FA (n=44)<br>4000 µg FA (n=43)<br>8000 µg FA (n=45)	32.9 (17.9, 28.4)[9,17]<br>53.6 (50.6, 64.2)[9,17]<br>77.0 (72.9, 86.7)[9,17]	Unavailable	Unavailable
**Title, L. 2000 [84]**	Double-blind, randomized controlled trial	50 participants<br>Age:18-75[7]<br>Novia Scotia, Canada	5 mg FA/d or 5 mg FA/d + 2g Vit C/d for 4 mo	Plasma folate	Immunoassay (Abbott IMX)	**Baseline:**<br>5000 µg FA (n=25)<br>5000 µg FA + 2g Vit C (n=25)	13.8 (11.8, 16.1)[17]<br>14.9 (13.3, 16.8)[17]	Unavailable	Unavailable
						**Intervention: 4mo**<br>5000 µg FA (n=25)<br>5000 µg FA + 2g Vit C (n=25)	79.2 (49.5, 126.7)[17]<br>80.1 (53.5, 119.9)[17]	Unavailable	Unavailable
**Toprak, A. 2005 [85]**	Randomized control trial	55 non-hysterectomized healthy postmenopausal females<br>Istanbul, Turkey	Hormone therapy (HT) + 5 mg FA/d for 3 mo<br><br>HT was 0.625 mg conjµgated equine estrogen, continuously combined with 2.5 mg medroxyprogesterone acetate daily	Serum folate	Electrochemiluminescence Immunoassay (Elecsys 2010, Roche Diagnostics)	**Baseline:**<br>HT + 5 mg/d FA: (n=20)	21.57 ± 9.53[5,9]	Unavailable	Unavailable
						**Intervention: 3 mos**<br>HT + 5 mg/d FA: (n=20)	40.41 ± 7.95[5,9]	Unavailable	Unavailable
**Tsui, JC. 1990 [86]**	Dietary intervention trial	23 adolescents<br>Age: 12-15[7]<br>Kansas City, Missouri, USA	400 µg FA/d for 8 wks	Serum folate	Radioassay	**Baseline:**<br>400 µg FA/d (n=23)	18.4 ± 8.1[6]	Unavailable	Unavailable
						**Intervention: 8wks**<br>400 µg FA/d (n=23)	29.2 ± 12.9[6]	Unavailable	Unavailable
**Tucker, K. 2004 [87]**	Randomized, double-blind trial	189 healthy, volunteers<br>Age: 50-85[7]	Daily consumption of breakfast cereal fortified with 440 µg FA or placebo for 12 weeks	Plasma folate	Radioassay (Bio-Rad, California, USA)	**Baseline:**<br>440 µg/d FA (n=93)	24.7 ± 0.7[6]	Unavailable	375 ± 17[6]
						**Intervention: 12 weeks**<br>440 µg/d FA (n=93)	32.2 ± 0.7[6]	Unavailable	745 ± 14[6]
**Ubbink, J. 1994 [88]**	Randomized controlled trial	100 males with hyperhomocysteinemia (>16.3 µmol/L)<br>Age: 20-73[7]<br>Pretoria, South Africa	0.65 mg FA/d for 6 wks or<br><br>0.65 mg FA/d + 12.2 mg Pyridoxine + 0.4 mg Cobalamin for 6 wks	Plasma folate	Radioimmunoassay (Simul TRAC-SNB, Becton Dickinson, New York, USA)	**Baseline:**<br>0.65 mg FA/d: (n=19)<br><br>Combo/d: 0.65 mg FA + 12.2 mg Pyridoxine + 0.4 mg Cobalamin (n=20)	6.0 ± 4.4[5]<br><br>5.2 ± 3.0[5]	Unavailable	Unavailable
						**Intervention: 6 wks**<br>0.65 mg FA/d: (n=19)	17.4 ± 10.4[5]<br><br>18.4 ± 11.1[5]	Unavailable	Unavailable

						Combo/d: 0.65 mg FA + 12.2 mg Pyridoxine + 0.4 mg Cobalamin (n=20)			
**Ubbink, JB. 1993 [89]**	Dietary intervention trial	20 men<br>Age: 18-65[7]<br>South Africa	1000 µg FA/d for 6 wks	Plasma folate	Radioimmunoassay (Becton Dickinson, New York, USA)	**Baseline:**<br>1000 µg FA/d (n=20)	5.3 ± 1.4[5]	Unavailable	Unavailable
						**Intervention : 6 wks**<br>1000 µg FA/d (n=20)	13.1 ± 5.5[5]	Unavailable	Unavailable
**Ubbink, JB. 1993 [90]**	Double-blind, randomized, placebo-controlled trial	30 men<br>Age: 19-71[7]<br>South Africa	1000 µg FA/d for 6 wks	Plasma folate	Radioimmunoassay (Becton Dickinson, New York, USA)	**Baseline:**<br>1000 µg FA/d (n=15)	5.6 ± 3.3[5]	Unavailable	Unavailable
						**Intervention: 4 wks**<br>1000 µg FA/d (n=15)	27.4 ± 13.8[5]	Unavailable	Unavailable
						**Intervention: 6wks**<br>1000 µg FA/d (n=15)	23.6 ± 14.6[5]	Unavailable	Unavailable
						**Intervention: 8wks**<br>2000 µg FA/d (n=15)	39.3 ± 21.9[5]	Unavailable	Unavailable
**Ubbink, JB. 1995 [91]**	Dietary intervention trial	30 men<br>Age: 19-24[7]<br>Pretoria, South Africa	1000 µg FA/d for 6 wks	Plasma folate	Radioimmunoassay (Becton Dickinson, New York, USA)	**Baseline:**<br>Whites: 1000 µg FA/d (n=18)<br>Blacks: 1000 µg FA/d (n=12)	8.3 ± 2.5[5]<br>7.7 ± 3.7[5]	Unavailable	Unavailable
						**Intervention: 6 wks**<br>Whites: 1000 µg FA/d (n=18)<br>Blacks: 1000 µg FA/d (n=12)	27.4 ± 11.8[5]<br>24.6 ± 15.1[5]	Unavailable	Unavailable
**Undas, A. 1999 [92]**	Dietary intervention trial	17 volunteers (6 women, 11 men) with elevated fasting Plasma Hcy levels<br>Age: 22-60[7]	5 mg FA/d for 8 weeks	Plasma foalte	Radioimmunoassay (Becton Dickinson, New York, USA)	**Baseline:**<br>5 mg FA/d (n=17)	8.6 (3.1, 18.9)[9,11]	Unavailable	Unavailable
						**Intervention: 8 wks**<br>5 mg FA/d (n=17)	55.8 (32.0, 66.3)[9,11]	Unavailable	Unavailable
**Venn, BJ. 2002 [93]**	Double-blind, randomized placebo-controlled trial	104 women of childbearing<br>Age:18-49[7]<br>Dunedin, New Zealand	100 µg FA/d for 24 wk	RBC and Plasma folate	Microbiologic- *L. casei*	**Baseline:**<br>100 µg FA/d (n=31)	25.2 ± 11.5[5]	932 ± 311[5]	Unavailable
						**Intervention: 24 wks**<br>100 µg FA/d (n=31)	34.8[18]	1131.2[18]	Unavailable
**Venn, BJ. 2002 [94]**	Double blind, randomized placebo-controlled trial	70 males and females<br>Age: >18<br>Dunedin, New Zealand<br>June-September 1999	Daily consumption of fortified cereal for 100, 200 or 300 µg FA for 4 weeks	Serum folate	Chemiluminescence (ACS180, Ciba Corning E, Massachusetts, USA)	**Baseline:**<br>100 µg FA/d (n=19)<br>200 µg FA/d (n=22)<br>300 µg FA/d (n=14)	18 (14, 22)[19]<br>17 (14, 20)[19]<br>18 (15, 22)[19]	Unavailable	Unavailable
						**Intervention: 4 wks**<br>100 µg FA/d (n=19)<br>200 µg FA/d (n=22)<br>300 µg FA/d (n=13)	23 (19, 28)[19]<br>28 (23, 33)[19]<br>33 (29, 38)[19]	Unavailable	Unavailable

**Venn, BJ. 2003 [95]**	Randomized, placebo-controlled study	167 healthy volunteers<br>Age: >18<br>Dunedin and Balclutha, New Zealand<br>March-April 2001	100 µg FA/d for 24 weeks or placebo	RBC and Plasma folate	Microbiologic- *L. casei*	**Baseline:**<br>100 µg FA/d (n=52)	23.3 (20.5, 26.5)[17]	915 (838, 999)[17]	211 (182, 244)[17]
						**Intervention: 8 wks**<br>100 µg FA/d (n=52)	28.9 (25.8, 32.4)[17]	999 (924, 1079)[17]	Unavailable
						**Intervention: 16 wks**<br>100 µg FA/d (n=52)	28.5 (24.6, 33.1)[17]	1057 (959, 1164)[17]	Unavailable
						**Intervention: 24 wks**<br>100 µg FA/d (n=52)	34.5 (30.5, 39.0)[17]	1137 (1053, 1227)[17]	Unavailable
**Vermeulen, EGJ. 2000 [96]**	Randomized, placebo-controlled trial	158 healthy siblings of 167 patients with premature atherothrombotic disease.<br>Netherlands<br>April 1993-January 1995	5 mg FA/d for 2 years	Plasma folate	Radioassay (Becton Dickinson, New York, USA)	**Baseline:**<br>5 mg FA/d (n=78)	12.1 ± 10.1[5]	Unavailable	Unavailable
						**Intervention: 1 year**<br>5 mg FA/d (n=72)	164.6 ± 229.1[5]	Unavailable	Unavailable
						**Intervention: 2 year**<br>5 mg FA/d (n= 68)	200.7 ± 300.9[5]	Unavailable	Unavailable
**Vliet, T. 2007 [97]**	Randomized, double-blinded, placebo-controlled, parallel trial	150 healthy volunteers<br>Age: 18-66[7]<br>Netherlands	Intervention: 6 weeks<br>200 µg FA/d or 400 µg FA/d	Serum folate	Competitive protein binding (Simultrac radioassay, ICN Pharmaceuticals Diagnostic, New York, USA)	**Baseline, male:**<br>200 µg FA/d (n=27)<br>400 µg FA/d (n=24)	 15.4 ± 6.0[5]<br>15.8 ± 3.5[5]	Unavailable	 196 ± 53[5]<br>220 ± 55[5]
						**Intervention 4 wks, male:**<br>200 µg FA/d (n=27)<br>400 µg FA/d (n=24)	 21.3[18]<br>26.9[18]	Unavailable	Unavailable
						**Intervention 6 wks, male:**<br>200 µg FA/d (n=27)<br>400 µg FA/d (n=24)	 22.9[18]<br>30.3[18]	Unavailable	Unavailable
						**Baseline, female :**<br>200 µg FA/d (n=22)<br>400 µg FA/d (n=24)	 16.1 ± 5.7[5]<br>13.7 ± 5.0[5]	Unavailable	 178 ± 54[5]<br>186 ± 61[5]
						**Intervention 4 wks, female:**<br>200 µg FA/d (n=21)<br>400 µg FA/d (n=24)	 22.8[18]<br>28.5[18]	Unavailable	Unavailable
						**Intervention 6 wks, female:**<br>200 µg FA/d (n=22)<br>400 µg FA/d (n=24)	 25.5[18]<br>33.7[18]	Unavailable	Unavailable
**Von Der Porten, AE. 1992 [98]**	Dietary intervention trial	6 Caucasian men<br>Age: 22-31[7]	1600 µg FA/d for 4 wks	Serum and RBC folate	Microbiologic- *L. casei*	**Baseline:**<br>1600 µg FA/d (n=6)	18[18]	501[18]	Unavailable
						**Intervention: 4 wks**<br>1600 µg FA/d (n=6)	45[18]	750[18]	Unavailable

**Voutilainen, S. 2003 [99]**	Double-blind, randomized controlled trial	20 healthy males<br>Age: 25.2 ± 3.8[5]<br>Kuopio, Finland	900 µg FA/d for 12 wks	Plasma folate	Radioimmunoassay (Quanta phase II, Bio-Rad, California, USA)	**Baseline:**<br>900 µg FA/d (n=20)	8.8 ± 3.3[5]	Unavailable	Unavailable
						**Intervention: 12 wks**<br>900 µg FA/d (n=20)	34.9 ± 25.6[23]	Unavailable	Unavailable
**Wald, DS. 2001 [100]**	Randomized, placebo-controlled trial	151 patients with ischemic heart diesease<br>Chichester, England	0.2, 0.6, 0.8 or 1.0 mg/d for 3 mo	Serum folate	Microbiologic- *L. casei*	**Baseline:**<br>Overall (n=151, includes placebo)<br>0.2 mg FA/d (n=27)<br>0.4 mg FA/d (n=25)<br>0.6 mg FA/d (n=25)<br>0.8 mg FA/d (n=25)<br>1.0 mg FA/d (n=24)	15.4[24], 18.4[18]	Unavailable	Unavailable
						**Intervention: 3 mo**<br>0.2 mg FA/d (n=26)<br>0.4 mg FA/d (n=24)<br>0.6 mg FA/d (n=25)<br>0.8 mg FA/d (n=25)<br>1.0 mg FA/d (n=24)	10.2[25]<br>26.1[25]<br>31.5[25]<br>46.0[25]<br>55.3[25]	Unavailable	Unavailable
**Wang, L. 2015 [101]**	Double-blind, randomized controlled trial	390 healthy participants<br>Age: 60-74[7]<br>Hebei province, China<br>July 2007-September 2012	400 µg FA for 12 months	Plasma folate	Microbiologic- *L. casei*	**Baseline:**<br>400 µg FA (n=195)	9.9 (9.2, 10.6)[17]	Unavailable	Unavailable
						**Intervention: 6 mo**<br>400 µg FA (n=180)	38.6[26]	Unavailable	Unavailable
						**Intervention: 12 mo**<br>400 µg FA (n=173)	37.2[26]	Unavailable	Unavailable
**Ward, M. 1997 [102]**	Crossover, dietary intervention trial	30 healthy male volunteers,<br>Age: 34-65[7]<br>Londenderry, Northern Ireland	100 µg FA/d for 6 wks; 200 µg FA/d for next 6 wks; 400 µg FA/d for next 14 weeks	Serum and RBC folate	Microbiologic- *L. casei*	**Baseline:**<br>baseline (n=30)	17.5 ± 10.4[5,9]	1060.1 ± 385.9[5]	Unavailable
						**Intervention: 6 wks**<br>100 µg FA/d (n=30)	20.7 ± 7.9[5,9]	Unavailable	Unavailable
						**Intervention: 12 wks**<br>200 µg FA/d (n=30)	25.2 ± 7.9[5,9]	Unavailable	Unavailable
						**Intervention: 26 wks**<br>400 µg FA/d (n=30)	39.0 ± 10.9[5,9]	1411.9 ± 397.3[5]	Unavailable
**Williams, E. 2005 [103]**	Placebo-controlled, crossover trial	10 males<br>Age: 32[18]<br>Ulster, Northern Ireland	100 µg/d FA for 6 weeks	RBC and Serum folate	Microbiologic- *L. casei*	**Baseline:**<br>100 µg/d FA (n=10)	9.9 ± 7.3[5]	969.3 ± 525.9[5]	Unavailable
						**Intervention: 6 weeks**<br>100 µg/d FA (n=10)	10.6[18]	1405.5[18]	Unavailable
**Winkels, R. 2008 [104]**	Randomized control trial	143 healthy males and females<br>Age: 50-75[7]<br>Netherlands	Daily consumption of fortified bread for 138 µg FA/day for 12 wks	RBC and Serum folate	Chemiluminescence immuoassay (Access Immunoassay, Beckman Coulter)	**Baseline:**<br>138 µg FA/day	15.7 ± 6.0[5]	n/a[10]	Unavailable

						**Intervention: 12 wks** 138 µg FA/day	20.2 ± 6.6[5]	n/a[10]	Unavailable
**Wolters, M. 2005 [105]**	Randomized control trial	220 healthy females Age: 60-91[7] Hanover , Germany	Daily consumption of multivitamin that contains 400 µg FA for 6 mo	RBC and Serum folate	Chemiluminescence system (ACS:180, Chiron Diagnostics, Fernwald, Germany)	**Baseline:** Multivitamin group (400µg FA/d) (n=108)	20.3 (12.5, 34.3)[27]	n/a	317 ± 86.2[5]
						**Intervention: 6 mos** Multivitamin group (400µg FA/d) (n=92)	58.9 (35.0, 89.5)[27]	n/a	Unavailable
**Woo, KS. 2002 [106]**	Dietary intervention trial	29 healthy adults with hyperhomocysteineimia, Age: 52± 8[5] Hong Kong	10 mg FA/d for 1 year	Plasma folate	Immunoassay (DPC dual-count solid-phase no-boil kit, Diagnostics Products Corporation, California, USA)	**Baseline:** 10 mg FA/d (n=29)	24 ± 5[5]	Unavailable	Unavailable
						**Intervention: 1 year** 10 mg FA/d (n=29)	40 ± 5[5]	Unavailable	Unavailable
**Wright, A. 2010 [107]**	Placebo-controlled Intervention Trial	163 healthy males and females Age: 18-65 United Kingdom	**Intervention:** Natural food folate and 200µg/d FA supplement feeding study (16 wks)	RBC and Plasma folate	Microbiologic- *L. casei*	**Baseline:** FA: (n=40)	24.1 ± 2.4[6]	833 ± 56[6]	711 ± 36[6]
						**Intervention: 16wks** FA: (n=40)	43.06[18]	1,154[18]	666.6 ± 30.8[6]
**Zheng, S. 1989 [108]**	Dietary intervention trial	150 healthy subjects Linxian, China	400 µg FA/d for 16 wks	RBC folate	Microbiologic- *L. casei*	**Baseline:** 400 µg FA/d (n=100)	Unavailable	449.5 ± 193.0[5,9]	Unavailable
						**Intervention: 16wks** 400 µg FA/d (n=88)	Unavailable	735.5 ± 340.5[5,9]	Unavailable

[1]Excepting unit conversions, all data in tables are as published in the literature or as provided by authors in correspondence. Data included in the meta-analyses may differ as discussed in the statistical methods.
[2]FA, folic acid; mo, months; wks, weeks; MZ, monozygotic; DZ, dizygotic
[3]Population characteristics (if given) as presented in the original paper (unless noted otherwise); sample size; age (in years); sampling description; ethnicity/nationality; study location; data collection periods
[4]Blood folate assay method: assay method; organism (if relevant); assay name (if given); manufacturer (if given)
[5]Mean ± SD
[6]Mean ± SEM
[7]Range
[8]Median (interquartile range)
[9]Values reported in µg/L or ng/mL and converted to nmol/L using: 1 µg/L=2.226 nmol/L
[10]Paper presented RBC folate data, but blood folate assay method used was not microbiologic
[11]Median (range)
[12]Mean increase from baseline (95% confidence interval)
[13]Median change from baseline (IQR)
[14]Geometric mean (range)
[15]Mean ± SD (geometric mean)
[16]Mean (95% confidence interval)
[17]Geometric mean (95% confidence interval)
[18]Mean
[19]Geometric mean (geometric SD)

[20]Least square mean (95% confidence interval)
[21]Geometric mean (minimum-maximum)
[22]Mean (interquartile range)
[23]Mean increase from baseline (SD)
[24]Median
[25]Median change from baseline
[26]Geometric mean
[27]Median (5th, 95th percentil

Table S2. Risk of bias (ROB) assessment tool questions by study design, as adapted from the *Item Bank for Assessment of Risk of Bias and Precision for Observational Studies*

Question followed by criteria for ranking each item as low, medium or high risk of bias	Cohort	Case-Control	Cross-sectional
**SELECTION BIAS**			
Clear inclusion/exclusion criteria:<br>*1. Does the article clearly state its own inclusion/exclusion criteria (i.e., does not require the reader to infer)?*<br><br>**Low**: Clearly states and defines (as appropriate) excluded groups<br>**Moderate**: Criteria partially stated or stated but not adequately defined (e.g., states that only healthy subjects are included but does not define healthy)<br>**High**: Criteria not stated	X	X	X
Appropriate sample selection:<br>*2. Is the sample appropriate?*<br><br>**Low**: Exclusion of unhealthy individuals and population-based<br>**Moderate**: —Exclusion of unhealthy individuals but not population-based<br>**High**: Non-exclusion of unhealthy individuals and not population-based	X	X	X
**DETECTION BIAS**			
Valid assessment of inclusion/exclusion:<br>*3. Are the inclusion/exclusion criteria measured using valid and reliable measures?*<br><br>**Low**: Physical exam, lab test, or other measure to verify "healthy, pregnancy status, or WCBA " status; survey of nationally-representative sample<br>**Moderate**: Self-report (questionnaire or interview); stated as healthy population but undefined<br>**High**: Inappropriate tool used in assessment of criteria	X	X	X
Valid assessment of exposure:<br>*4. Is the level of detail in describing the intervention or exposure adequate?*<br><br>**Low**: Provided intervention/intake information assessment method (i.e., dose, frequency) and included pill count or other compliance estimates, e.g., percent of compliance<br>**Moderate**: Provided intervention/intake information assessment method (i.e., dose, frequency); mentioned that they monitor pill use or other compliance estimates but did not include the percent of compliance in the manuscript<br>**High**: Included dose/frequency but no information provided regarding compliance assessment method	X	X	X
Valid assessment of outcome:<br>*5. Are outcomes (blood folate concentrations) assessed using valid and reliable measures, implemented consistently across all study participants?*<br><br>**Low**: Microbiologic assay for serum/plasma and/or RBC folate<br>**Moderate**: Any protein-binding assay for serum/plasma folate.<br>**High**: Protein-bind assay for RBC folate; unknown assay<br><br>*6. Are sample storage conditions appropriate?*	X	X	X

Question followed by criteria for ranking each item as low, medium or high risk of bias	Cohort	Case-Control	Cross-sectional
**Low:** If storage T ≤ -70ºC<br><br>**High:** If storage > -70ºC or storage temperature not specified			
**ATTRITION BIAS**			
Incomplete outcome data:<br><br>7. *Is attrition >20%?*<br><br>**Low**: No<br>**Moderate**: Attrition >20% but paper provides description of participants lost to follow-up<br>**High**: Attrition >20% and paper provides no description of participants lost	X	N/A	N/A

Table S3. Summary risk of bias tables for randomized controlled trials using Cochrane tool[1] and for non-randomized controlled trials and observational studies using adapted RTI item bank.

First Author	Selection bias		Detection bias				Attrition bias	Overall Risk of Bias[1]
Domains / Papers	Clear inclusion/ exclusion criteria[2]	Appropriate sample selection	Valid assessment of: Inclusion/ exclusion	Exposure[2]	Outcome for assay[2]	Outcome for storage[2]	Incomplete outcome data	
Aarabi, M. 2015	L	M	M	H	L	H	L	H
Adank, C. 2003	L	M	L	M	L	L	L	M
Amatayakul, K. 1984	L	M	M	M	M	H	L	H
Anderson, C. 2010	L	M	M	L	M	L	L	M
Anderson. C. 2013	L	M	M	L	M	H	H	H
Ashfield-Watt, P. 2001	L	M	L	M	M	H	L	H
Ashfield-Watt, P. 2002	L	M	L	H	M	L	L	H
Baro, L. 2003	L	M	L	M	M	L	L	M
Baro, L. 2004	L	M	L	M	M	L	H	H
Basoglu, C. 2009	L	M	L	H	M	H	L	H
Biswas, A. 2009	L	M	L	H	M	L	L	H
Bramswig, S. 2009	L	M	L	L	L	H	L	H
Bronstrup, A. 1998	H	H	H	M	M	H	L	H
Brouwer, I. 1998	L	M	L	M	M	H	L	H
Brouwer, I. 1999	L	M	M	L	M	H	L	H
Brouwer, I. 2000	L	M	M	M	M	H	L	H

Carlsson, CM. 2004	L	M	M	L	M	H	M	H
Cheong, M. 2016	L	M	L	L	L	L	L	M
Cotlarciuc, I. 2011	L	M	M	M	M	H	L	H
Cuskelly, GJ. 1996	L	M	M	M	L	H	H	H
Daly, S. 2002	L	M	L	L	L	H	M	H
Davis, BA. 1995	L	M	L	M	L	H	L	H
Deshmukh, U. 2010	L	M	L	L	L	L	L	M
Dierkes, J. 1998	L	M	L	L	M	H	L	H
Doshi, S. 2003	L	M	L	H	M	H	L	H
Doshi, SN. 2002	L	M	L	L	M	L	L	M
Dragani, A. 2010	L	M	L	M	M	L	L	M
Durga, J. 2007	L	M	L	L	M	L	L	M
Duthie, S. 2010	L	M	L	H	M	H	L	H
Earnest, C. 2002	M	H	M	H	L	L	L	H
Ebisch, IMW. 2006	L	M	L	L	M	H	L	H
Fohr, I. 2002	L	M	L	L	M	H	L	H
Gamble, M. 2006	H	H	M	L	M	L	L	H
Green, T. 2005	L	M	M	L	L	L	L	M
Green, T. 2013	L	M	L	M	L	L	L	M
Green, TJ. 2003	L	M	L	H	L	H	L	H
Grieger, J. 2009	L	M	M	M	M	L	L	M
Haglund, O. 1993	L	M	L	M	M	L	L	M
Hao, L. 2008	L	L	L	L	L	L	L	L
Herrmann, M. 2006	M	M	M	H	M	H	L	H
Heseker, H. 1987	M	M	M	H	M	H	L	H
Heseker, H. 1993	M	M	M	H	M	L	H	H
Hiraoka, M. 2004	M	M	M	H	M	H	L	H
Holmes, T. 2003	L	M	M	M	M	H	L	H
Hursthouse, N. 2011	L	M	M	L	L	L	L	M
Johansson, M. 2002	L	M	L	H	M	H	L	H

Kauwell, G. 2000	L	M	L	L	L	H	L	H
Klerk, M. 2004	L	M	L	L	M	L	L	M
Kok, DEG. 2015	L	L	L	H	M	H	L	H
Lamers, Y. 2004	L	L	L	L	M	H	L	H
Liem, A. 2003	L	M	L	H	M	H	L	H
Lin, P. 2006	L	M	L	L	M	H	L	H
Mann, BA. 1987	H	H	H	M	M	L	H	H
Mao, G. 2008	L	M	L	M	M	L	L	M
Martin-Bautista, E. 2010	L	M	L	L	M	L	L	M
McKay, DL. 2000	L	L	L	L	M	L	M	M
Meer, K. 2005	L	M	L	L	M	L	L	M
Melse-Boonstra, A. 2004	L	M	L	L	M	L	L	M
Nguyen, P. 2008	L	L	L	L	L	L	L	L
Nguyen, P. 2009	L	M	L	L	L	L	L	M
Norsworthy, B. 2001	L	M	L	M	L	H	L	H
Olthof, M. 2006	L	M	L	L	M	L	L	M
Oort, F. 2003	L	M	L	L	M	L	L	M
Papandreou, D. 2010	H	H	L	H	M	H	L	H
Pathansali, R. 2006	L	M	L	H	M	H	L	H
Pena, A. 2007	L	M	L	L	M	H	L	H
Petrogianni, M. 2012	L	M	L	M	M	L	L	M
Pinto, X. 2005	L	M	L	M	M	H	L	H
Pullin, CH. 2001	L	M	L	M	M	L	L	M
Qin, X. 2012	L	M	L	L	M	L	L	M
Quinlivan, EP. 2002	L	M	L	H	L	H	L	H
Raigani, M. 2014	L	M	L	H	M	H	L	H
Rhode, BM. 1983	L	M	M	M	L	H	L	H
Rosenthal, J. 2008	L	M	L	M	M	H	M	H
Schorah, CJ. 1998	L	M	M	H	M	H	L	H
Sharifi, F. 2010	L	L	L	H	M	H	L	H

Sheu, W. 2005	L	M	L	L	M	L	L	M
Shidfar, F. 2009	L	M	L	H	M	H	L	H
Smidt, LJ. 1990	L	L	L	L	L	H	L	H
Strandhagen, E. 2003	L	M	L	H	M	L	L	H
Sweeney, MR 2007	L	M	M	M	L	H	L	H
Tapola, NS. 2004	L	L	L	L	M	H	L	H
Tighe, T. 2011	L	M	L	L	L	H	L	H
Title, L. 2000	L	M	L	L	M	L	L	M
Toprak, A. 2005	L	M	L	L	M	H	L	H
Tsui, JC. 1990	L	M	L	L	M	H	M	H
Tucker, K. 2004	L	M	M	L	M	L	L	M
Ubbink, J. 1994	L	M	L	M	M	L	L	M
Ubbink, JB. 1993	L	M	L	H	M	H	L	H
Ubbink, JB. 1993	L	M	L	H	M	H	L	H
Ubbink, JB. 1995	L	M	L	L	M	H	L	H
Undas, A. 1999	L	M	L	H	M	L	L	H
Venn, BJ. 2002	L	M	M	M	L	H	L	H
Venn, BJ. 2002	L	M	L	M	L	H	L	H
Venn, BJ. 2003	L	L	M	L	L	L	L	M
Vermeulen, EGJ. 2000	L	L	L	L	M	L	L	M
Vliet, T. 2007	L	M	L	L	M	H	L	H
Von Der Porten, AE. 1992	L	M	L	M	L	H	L	H
Voutilainen, S. 2003	L	M	L	M	M	L	L	M
Wald, DS. 2001	L	M	M	L	L	H	L	H
Wang, L. 2015	L	M	M	L	L	L	L	M
Ward, M. 1997	L	M	M	M	L	H	L	H
Williams, E. 2005	L	M	L	M	L	L	L	M
Winkels, R. 2008	L	M	L	L	M	L	L	M
Wolters, M. 2005	L	M	M	M	M	H	L	H
Woo, KS. 2002	L	M	M	L	M	L	L	M

Wright, A. 2010	L	M	L	H	L	H	L	H
Zheng, S. 1989	H	M	H	L	L	H	L	H

[1] Risk of bias assessment: L = low; M = moderate; H = high

[2] Cochrane recommends stating potential sources of bias not addressed in the other domains of the risk of bias tool. Additional potential sources of bias identified in our systematic review include clear inclusion/exclusion criteria across comparison groups, and valid assessments for exposure and outcome (assay used and sample storage conditions)

Protocol: Folic acid supplementation and blood folate concentration – a systematic review and meta-analysis

Authors
Krista S Crider, Owen J Devine, Ahlia Sekkarie, Ibrahim Zaganjor, Yan Ping Qi, Robert J Berry

National Center on Birth Defects and Developmental Disabilities, Centers for Disease Control and Prevention, Atlanta, Georgia, USA

Research question
How does folic acid intake change blood folate concentrations?

Objective
To perform a systematic review and meta-analysis of impact of folic acid supplementation on and blood folate concentrations

Methods
Search strategy
A research librarian from the CDC Public Health Library will be consulted and will perform the literature search using the following search terms (adapted for databases as necessary) for English language literature published between January 1980 - October 2014.

We will use the following search strategy to search Embase and adapt as required for other electronic databases:

1. Folic acid.mp or folic acid/
2. Blood folate.mp or exp folic acid blood level/
3. (Serum folate or plasma folate or red blood cell folate or erythrocyte folate).mp
4. (Intake or diet* or supplement*).mp
5. Folic acid intake.mp
6. Limit 21 to (English language and yr=”1980–October 2014”)

In addition to Embase, the following databases will be searched: Cochrane Library, CINAHL, POPLINE, PubMed, and Web of Science

To identify additional resources, we will search the references of all articles in the full text review.

If supplement use stratified data or other population information is needed for eligibility in the review, we will contact authors for additional information.

Inclusion criteria

- All studies will be considered, with the exception of case studies and cross sectional studies:
    - Randomized trials, quasi-randomized, and non-randomized control trials with individual or cluster level randomization.
    - Supplementation trials without placebo control will be included (although evaluated in sensitive analysis.)

Exclusion criteria

- Studies that do not contain information on the relationship between at least two of the four following outcome measures: e.g., must be able to calculate change in blood folate concentrations (concentration after XX days/weeks/months/years - baseline concentration – before known dose of folic acid was administered).
    - Serum/plasma folate or red blood cell (RBC), erythrocyte folate at baseline and during intervention
    - Folic acid intake dose

- Studies including unhealthy populations, defined as sample recruitment based on a health condition.
    - Including, but not limited to: conditions impacting folate absorption, such as intestinal malabsorption conditions, inflammatory bowel disease, alcohol abuse, cancer, chronic congestive heart failure, chronic renal failure, chronic liver failure, or chronic infectious disease.
- Studies in pregnant, lactating.
- Studies without blood folate concentrations stratified by folic acid containing supplement use.

Selection of studies

Excel will be used to store all identified studies from the search strategy. Titles and abstracts will be screened in duplicate. Three teams of two review abstracts and full text files (KSC+YPQ, YPQ+IZ, and KSC+AS).

Data extraction

For eligible studies, two reviewers will independently extract 1/3 of data using a piloted abstraction form. Any discrepancies will be resolved through discussion and document the process.

The following information will be extracted from each study:

1. Methods
    - Study design
    - Folate assay method
2. Participants
    - Location of the study
    - Data collection time frame
    - Sample size
    - Age
    - Blood folate concentrations
    - Natural food folate intake
    - Method of dietary assessment
3. Intervention
    - Folic acid dose
    - Folate intake if provided/reported
    - Duration of the intervention specify for each data point

Risk of bias
Two review authors (YPQ, KSC) developed and adapted tools for the assessment of risk of bias. For control trials, the *Cochrane Handbook for Systematic Reviews of Interventions* was used [109]. For observational studies, an adapted Item Bank on Risk of Bias and Precision of Observational Studies from the Research Triangle Institute (RTI) was used [110]. LFY and KSC will independently evaluate risk of bias for each study for the domains below.

Domain risk of bias
A score of low, moderate, and high risk of bias score will be assigned to each domain for each study outcome (i.e. serum/plasma folate and RBC folate concentrations). Domains will be considered low risk of bias if all questions within a domain are graded as low, moderate risk of bias if one or more questions are graded as moderate or high risk of bias if one or more questions are graded as high. If we are unable to determine a domain's risk of bias due to insufficient information provided in a study, the domain will be classified as "unclear" in the risk of bias tables.

Overall risk of bias
This same convention will be applied across domains to assign an overall summary score of low, moderate, or high risk of bias for each study outcome (i.e., serum/plasma folate and RBC folate concentrations).

Control trial domains:
- Random sequence generation
- Allocation concealment
- Blinding of participants and personnel
- Blinding of outcome assessment
- Incomplete outcome bias
- Reporting bias
- Other sources of bias (as needed)

Observational study domains:
- Uniform and valid measures of inclusion/exclusion criteria
- Appropriateness of study sample
- Exposure and outcome assessment methods
- Appropriate follow-up time[1]
- Completeness of outcome data[1]
- Adjustment for potential confounding

Data analysis
Meta-analyses
- A statistician (OJD) will use Open BUGS 3.2.2 software as statistical software.
- A Bayesian Markov Monte Carlo chain procedure will be used to develop posterior distributions

[1] Appropriate follow-up time and completeness of outcome data will not be assessed for cross-sectional studies.

    - Estimates of change over doses of folic acid between 100 µg/day to 4000 µg/day are anticipated. Changes at 4, 12, and 24 weeks of supplementation will be models.
    - Dose-response curves will then be extrapolated to a homoeostatic state (steady state) for each individual dose and those used to create a steady state response.
    - The steady state response will be compared to estimates from cross-sectional studies of fortified countries in post hoc analysis (e.g. NHANES US)
- A Bayesian Markov Monte Carlo chain procedure will be used to develop posterior distributions.
    - Each study will be treated as a random effect.
    - The percent change in blood folate concentrations for every doubling of natural food folate intake will be calculated.
- Sensitivity analyses performed will depend on the number of studies eligible for inclusion in the meta-analyses. Anticipated sensitivity analyses of the association of natural food folate intake and blood folate concentrations may be stratified by:
    - Study quality (risk of bias, study tiers)
    - Assay type
    - Study design
    - Country fortification policy status
    - Baseline folate status

References

1. Aarabi, M.; San Gabrie, M.C.; Chan, D.; Behan, N.A.; Caron, M.; Pastinen, T.; Bourque, G.; MacFarlane, A.J.; Zini, A.; Trasler, J. High-dose folic acid supplementation alters the human sperm methylome and is influenced by the mthfr c677t polymorphism. *Human Molecular Genetics* **2015**, *24*, 6301-6313.
2. Adank, C.; Green, T.J.; Skeaff, C.M.; Briars, B. Weekly high-dose folic acid supplementation is effective in lowering serum homocysteine concentrations in women. *Annals of Nutrition & Metabolism* **2003**, *47*, 55-59.
3. Amatayakul, K.; Uttaravichai, C.; Singkamani, R.; Ruckphaopunt, S. Vitamin metabolism and the effects of multivitamin supplementation in oral contraceptive users. *Contraception* **1984**, *30*, 179-196.
4. Anderson, C.A.; Jee, S.H.; Charleston, J.; Narrett, M.; Appel, L.J. Effects of folic acid supplementation on serum folate and plasma homocysteine concentrations in older adults: A dose-response trial. *American Journal of Epidemiology* **2010**, *172*, 932-941.
5. Anderson, C.A.; Beresford, S.A.; McLerran, D.; Lampe, J.W.; Deeb, S.; Feng, Z.; Motulsky, A.G. Response of serum and red blood cell folate concentrations to folic acid supplementation depends on methylenetetrahydrofolate reductase c677t genotype: Results from a crossover trial. *Molecular Nutrition & Food Research* **2013**, *57*, 637-644.
6. Ashfield-Watt, P.A.; Knowles, R.M.; Cale, S.B.; Payne, N.; McDowell, I.F.W. A folate-enriched diet lowers plasma homocysteine in patients with hyperlipidaemia: A randomised controlled trial. *British Journal of Cardiology* **2001**, *8*, 20-30+32-35.
7. Ashfield-Watt, P.A.; Pullin, C.H.; Whiting, J.M.; Clark, Z.E.; Moat, S.J.; Newcombe, R.G.; Burr, M.L.; Lewis, M.J.; Powers, H.J.; McDowell, I.F. Methylenetetrahydrofolate reductase 677c-->t genotype modulates homocysteine responses to a folate-rich diet or a low-dose folic acid supplement: A randomized controlled trial. *American Journal of Clinical Nutrition* **2002**, *76*, 180-186.
8. Baro, L.; Fonolla, J.; Pena, J.L.; Martinez-Ferez, A.; Lucena, A.; Jimenez, J.; Boza, J.J.; Lopez-Huertas, E. N-3 fatty acids plus oleic acid and vitamin supplemented milk consumption reduces total and ldl cholesterol, homocysteine and levels of endothelial adhesion molecules in healthy humans. *Clinical Nutrition* **2003**, *22*, 175-182.
9. Baro, L.; Martinez-Ferez, A.; Rodriguez, C.; Valero, A.; Fonolla, J.; Lucena, A.; Jimenez, J.; Boza, J.J.; Lopez-Huertas, E. The administration of a multivitamin/mineral fortified dairy product improves folate status and reduces plasma homocysteine concentration in women of reproductive age. *International Journal for Vitamin & Nutrition Research* **2004**, *74*, 234-240.
10. Basoglu, C.; Ates, M.A.; Algul, A.; Ipcioglu, O.M.; Gecici, O.; Yilmaz, O.; Semiz, U.B.; Ebrinc, S.; Gulsun, M.; Ozcan, O.*, et al.* Adjuvant folate with escitalopram treatment and homocystein, folate, vitamin b-12 levels in patients with major depressive disorder. *Klinik Psikofarmakoloji Bulteni* **2009**, *19*, 135-142.
11. Biswas, A.; Ranjan, R.; Meena, A.; Akhter, M.S.; Yadav, B.K.; Munisamy, M.; Subbiah, V.; Behari, M.; Saxena, R. Homocystine levels, polymorphisms and the risk of ischemic stroke in young asian indians. *Journal of Stroke and Cerebrovascular Diseases* **2009**, *18*, 103-110.
12. Bramswig, S.; Prinz-Langenohl, R.; Lamers, Y.; Tobolski, O.; Wintergerst, E.; Berthold, H.K.; Pietrzik, K. Supplementation with a multivitamin containing 800 microg of folic acid shortens the time to reach the preventive red blood cell folate concentration in healthy women. *International Journal for Vitamin & Nutrition Research* **2009**, *79*, 61-70.
13. Bronstrup, A.; Hages, M.; Prinz-Langenohl, R.; Pietrzik, K. Effects of folic acid and combinations of folic acid and vitamin b-12 on plasma homocysteine concentrations in healthy, young women. *American Journal of Clinical Nutrition* **1998**, *68*, 1104-1110.
14. Brouwer, I.A.; Dusseldorp, M.; West, C.E.; Meyboom, S.; Thomas, C.M.G.; Duran, M.; het Hof, K.; Eskes, T.; Hautvast, J.; Steegers-Theunissen, R.P.M. High daily intake of dietary folate decreases homocysteine and improves folate status: A controlled dietary trial in young healthy volunteers. In *Netherlands journal of medicine*, 1998; Vol. 52, p S41.

15. Brouwer, I.A.; Dusseldorp, M.; Thomas, C.M.; Duran, M.; Hautvast, J.G.; Eskes, T.K.; Steegers-Theunissen, R.P. Low-dose folic acid supplementation decreases plasma homocysteine concentrations: A randomized trial. In *American journal of clinical nutrition*, 1999; Vol. 69, pp 99-104.
16. Brouwer, I.A.; Van Rooij, I.A.L.M.; Van Dusseldorp, M.; Thomas, C.M.G.; Blom, H.J.; Hautvast, J.G.A.J.; Eskes, T.K.A.B.; Steegers-Theunissen, R.P.M. Homocysteine-lowering effect of 500 mug folic acid every other day versus 250 mug/day. *Annals of Nutrition and Metabolism* **2000**, *44*, 194-197.
17. Carlsson, C.M.; Pharo, L.M.; Aeschlimann, S.E.; Mitchell, C.; Underbakke, G.; Stein, J.H. Effects of multivitamins and low-dose folic acid supplements on flow-mediated vasodilation and plasma homocysteine levels in older adults. *American Heart Journal* **2004**, *148*, E11.
18. Neuhouser, M.L.; Nijhout, H.F.; Gregory, J.F., 3rd; Reed, M.C.; James, S.J.; Liu, A.; Shane, B.; Ulrich, C.M. Mathematical modeling predicts the effect of folate deficiency and excess on cancer-related biomarkers. *Cancer Epidemiology, Biomarkers & Prevention* **2011**, *20*, 1912-1917.
19. Cotlarciuc, I.; Andrew, T.; Dew, T.; Clement, G.; Gill, R.; Surdulescu, G.; Sherwood, R.; Ahmadi, K.R. The basis of differential responses to folic acid supplementation. *Journal of Nutrigenetics & Nutrigenomics* **2011**, *4*, 99-109.
20. Cuskelly, G.J.; McNulty, H.; Scott, J.M. Effect of increasing dietary folate on red-cell folate: Implications for prevention of neural tube defects. *Lancet* **1996**, *347*, 657-659.
21. Daly, S.; Mills, J.L.; Molloy, A.M.; Conley, M.; McPartlin, J.; Lee, Y.J.; Young, P.B.; Kirke, P.N.; Weir, D.G.; Scott, J.M. Low-dose folic acid lowers plasma homocysteine levels in women of child-bearing age. *Qjm* **2002**, *95*, 733-740.
22. Davis, B.A.; Bailey, L.B.; Gregory, J.F., 3rd; Toth, J.P.; Dean, J.; Stevenson, R.E. Folic acid absorption in women with a history of pregnancy with neural tube defect. *American Journal of Clinical Nutrition* **1995**, *62*, 782-784.
23. Deshmukh, U.S.; Joglekar, C.V.; Lubree, H.G.; Ramdas, L.V.; Bhat, D.S.; Naik, S.S.; Hardikar, P.S.; Raut, D.A.; Konde, T.B.; Wills, A.K.*, et al.* Effect of physiological doses of oral vitamin b12 on plasma homocysteine: A randomized, placebo-controlled, double-blind trial in india. *European Journal of Clinical Nutrition* **2010**, *64*, 495-502.
24. Dierkes, J.; Kroesen, M.; Pietrzik, K. Folic acid and vitamin b6 supplementation and plasma homocysteine concentrations in healthy young women. *International Journal for Vitamin & Nutrition Research* **1998**, *68*, 98-103.
25. Doshi, S.; McDowell, I.; Moat, S.; Lewis, M.; Goodfellow, J. Folate improves endothelial function in patients with coronary heart disease. In *Clinical chemistry and laboratory medicine*, 2003; Vol. 41, pp 1505-1512.
26. Doshi, S.N.; McDowell, I.F.; Moat, S.J.; Payne, N.; Durrant, H.J.; Lewis, M.J.; Goodfellow, J. Folic acid improves endothelial function in coronary artery disease via mechanisms largely independent of homocysteine lowering. *Circulation* **2002**, *105*, 22-26.
27. Santilli, F.; Basili, S.; Dragani, A.; Falco, A.; Rolandi, G.; Cerasa, L.; Lattanzio, S.; Ciabattoni, G.; Patrono, C.; Davi, G. Oxidati ve stress and platelet activation in subjects with moderate hyperhomocysteinemia due to mthfr 677 c->t polymorphism. *Pathophysiology of Haemostasis and Thrombosis* **2010**, *37*, A56.
28. Durga, J.; Verhoef, P.; Anteunis, L.J.; Schouten, E.; Kok, F.J. Effects of folic acid supplementation on hearing in older adults: A randomized, controlled trial.[summary for patients in ann intern med. 2007 jan 2;146(1):I20; pmid: 17200213]. *Annals of Internal Medicine* **2007**, *146*, 1-9.
29. Duthie, S.J.; Horgan, G.; Roos, B.; Rucklidge, G.; Reid, M.; Duncan, G.; Pirie, L.; Basten, G.P.; Powers, H.J. Blood folate status and expression of proteins involved in immune function, inflammation, and coagulation: Biochemical and proteomic changes in the plasma of humans in response to long-term synthetic folic acid supplementation. In *Journal of proteome research*, 2010; Vol. 9, pp 1941-1950.
30. Earnest, C.; Cooper, K.H.; Marks, A.; Mitchell, T.L. Efficacy of a complex multivitamin supplement. *Nutrition* **2002**, *18*, 738-742.

31. Ebisch, I.M.W.; Pierik, F.H.; De Jong, F.H.; Thomas, C.M.G.; Steegers-Theunissen, R.P.M. Does folic acid and zinc sulphate intervention affect endocrine parameters and sperm characteristics in men? *International Journal of Andrology* **2006**, *29*, 339-345.
32. Fohr, I.P.; Prinz-Langenohl, R.; Brönstrup, A.; Bohlmann, A.M.; Nau, H.; Berthold, H.K.; Pietrzik, K. 5, 10-methylenetetrahydrofolate reductase genotype determines the plasma homocysteine-lowering effect of supplementation with 5-methyltetrahydrofolate or folic acid in healthy young women. *American Journal of Clinical Nutrition* **2002**, *75*, 275-282.
33. Gamble, M.V.; Liu, X.; Ahsan, H.; Pilsner, J.R.; Ilievski, V.; Slavkovich, V.; Parvez, F.; Chen, Y.; Levy, D.; Factor-Litvak, P*., et al.* Folate and arsenic metabolism: A double-blind, placebo-controlled folic acid-supplementation trial in bangladesh. *American Journal of Clinical Nutrition* **2006**, *84*, 1093-1101.
34. Green, T.J.; Skeaff, C.M.; Rockell, J.E.; Venn, B.J. Folic acid fortified milk increases blood folate and lowers homocysteine concentration in women of childbearing age. *Asia Pacific Journal of Clinical Nutrition* **2005**, *14*, 173-178.
35. Green, T.J.; Liu, Y.; Dadgar, S.; Li, W.; Böhni, R.; Kitts, D.D. Wheat rolls fortified with microencapsulated l-5-methyltetrahydrofolic acid or equimolar folic acid increase blood folate concentrations to a similar extent in healthy men and women. In *Journal of nutrition*, 2013; Vol. 143, pp 867-871.
36. Green, T.J.; Skeaff, C.M.; Whiting, S.J.; Gibson, R.S. Effect of folic acid supplementation on plasma zinc concentrations of young women. *Nutrition* **2003**, *19*, 522-523.
37. Grieger, J.A.; Nowson, C.A. Use of calcium, folate, and vitamin d3-fortified milk for 6 months improves nutritional status but not bone mass or turnover, in a group of australian aged care residents. *Journal of Nutrition for the Elderly* **2009**, *28*, 236-254.
38. Haglund, O.; Hamfelt, A.; Hambraeus, L.; Saldeen, T. Effects of fish oil supplemented with pyridoxine and folic acid on homocysteine, atherogenic index, fibrinogen and plasminogen activator inhibitor-1 in man. *Nutrition Research* **1993**, *13*, 1351-1365.
39. Hao, L.; Yang, Q.H.; Li, Z.; Bailey, L.B.; Zhu, J.H.; Hu, D.J.; Zhang, B.L.; Erickson, J.D.; Zhang, L.; Gindler, J*., et al.* Folate status and homocysteine response to folic acid doses and withdrawal among young chinese women in a large-scale randomized double-blind trial. *American Journal of Clinical Nutrition* **2008**, *88*, 448-457.
40. Herrmann, M.; Stanger, O.; Paulweber, B.; Hufnagl, C.; Herrmann, W. Folate supplementation does not affect biochemical markers of bone turnover. *Clinical Laboratory* **2006**, *52*, 131-136.
41. Heseker, H.; Schmitt, G. Effect of long-term supplementation of folate on folate status in plasma and erythrocytes. *Journal of Nutritional Science & Vitaminology* **1987**, *33*, 163-168.
42. Heseker, H.; Kubler, W. Chronically increased vitamin intake and vitamin status of healthy men. *Nutrition* **1993**, *9*, 10-17; discussion 68.
43. Hiraoka, M.; Kato, K.; Saito, Y.; Yasuda, K.; Kagawa, Y. Gene-nutrient and gene-gene interactions of controlled folate intake by japanese women. *Biochemical & Biophysical Research Communications* **2004**, *316*, 1210-1216.
44. Holmes, T.; Gates, G.E. The effect of fortified breakfast cereal on plasma homocyst(e)ine concentrations in healthy older men already consuming a folate fortified diet. *Nutrition Research* **2003**, *23*, 435-449.
45. Hursthouse, N.A.; Gray, A.R.; Miller, J.C.; Rose, M.C.; Houghton, L.A. Folate status of reproductive age women and neural tube defect risk: The effect of long-term folic acid supplementation at doses of 140 g and 400 g per day. *Nutrients* **2011**, *3*, 49-62.
46. Johansson, M.; Witthöft, C.M.; Bruce, Å.; Jägerstad, M. Study of wheat breakfast rolls fortified with folic acid: The effect of folate status in women during a 3-month intervention. *European Journal of Nutrition* **2002**, *41*, 279-286.
47. Kauwell, G.P.; Lippert, B.L.; Wilsky, C.E.; Herrlinger-Garcia, K.; Hutson, A.D.; Theriaque, D.W.; Rampersaud, G.C.; Cerda, J.J.; Bailey, L.B. Folate status of elderly women following moderate folate depletion responds only to a higher folate intake. *Journal of Nutrition* **2000**, *130*, 1584-1590.

48. Klerk, M.; Durga, J.; Schouten, E.G.; Kluft, C.; Kok, F.J.; Verhoef, P. No effect of folic acid supplementation in the course of 1 year on haemostasis markers and c-reactive protein in older adults. *Thrombosis & Haemostasis* **2005**, *94*, 96-100.
49. Kok, D.E.; Dhonukshe-Rutten, R.A.; Lute, C.; Heil, S.G.; Uitterlinden, A.G.; van der Velde, N.; van Meurs, J.B.; van Schoor, N.M.; Hooiveld, G.J.; de Groot, L.C.*, et al.* The effects of long-term daily folic acid and vitamin b12 supplementation on genome-wide DNA methylation in elderly subjects. *Clinical Epigenetics* **2015**, *7*, 121.
50. Lamers, Y.; Prinz-Langenohl, R.; Moser, R.; Pietrzik, K. Supplementation with [6s]-5-methyltetrahydrofolate or folic acid equally reduces plasma total homocysteine concentrations in healthy women. *American Journal of Clinical Nutrition* **2004**, *79*, 473-478.
51. Liem, A.; Reynierse-Buitenwerf, G.H.; Zwinderman, A.H.; Jukema, J.W.; Veldhuisen, D.J. Secondary prevention with folic acid: Effects on clinical outcomes. In *Journal of the American College of Cardiology*, 2003; Vol. 41, pp 2105-2113.
52. Lin, P.T.; Lee, B.J.; Chang, H.H.; Cheng, C.H.; Tsai, A.J.; Huang, Y.C. Low-dose folic acid supplementation reduces homocysteine concentration in hyperhomocysteinemic coronary artery disease patients. *Nutrition Research* **2006**, *26*, 460-466.
53. Mann, B.A.; Garry, P.J.; Hunt, W.C.; Owen, G.M.; Goodwin, J.S. Daily multivitamin supplementation and vitamin blood levels in the elderly: A randomized, double-blind, placebo-controlled trial. *Journal of the American Geriatrics Society* **1987**, *35*, 302-306.
54. Mao, G.; Xiumei, H.; Xing, H.; Liu, P.; Liu, H.; Yu, Y.; Zhang, S.; Jiang, S.; Wang, X.; Xu, Z. Efficacy of folic acid and enalapril combined therapy on reduction of blood pressure and plasma glucose: A multicenter, randomized, double-blind, parallel-controlled, clinical trial. *Nutrition* **2008**, *24*, 1088-1096.
55. Martin-Bautista, E.; Muñoz-Torres, M.; Fonolla, J.; Quesada, M.; Poyatos, A.; Lopez-Huertas, E. Improvement of bone formation biomarkers after 1-year consumption with milk fortified with eicosapentaenoic acid, docosahexaenoic acid, oleic acid, and selected vitamins. In *Nutrition research (New York, N.Y.)*, 2010; Vol. 30, pp 320-326.
56. McKay, D.L.; Perrone, G.; Rasmussen, H.; Dallal, G.; Blumberg, J.B. Multivitamin/mineral supplementation improves plasma b-vitamin status and homocysteine concentration in healthy older adults consuming a folate-fortified diet. *Journal of Nutrition* **2000**, *130*, 3090-3096.
57. Meer, K.; Smulders, Y.M.; Dainty, J.R.; Smith, D.E.; Kok, R.M.; Stehouwer, C.D.; Finglas, P.M.; Jakobs, C. [6s]5-methyltetrahydrofolate or folic acid supplementation and absorption and initial elimination of folate in young and middle-aged adults. In *European journal of clinical nutrition*, 2005; Vol. 59, pp 1409-1416.
58. Melse-Boonstra, A.; West, C.E.; Katan, M.B.; Kok, F.J.; Verhoef, P. Bioavailability of heptaglutamyl relative to monoglutamyl folic acid in healthy adults. *American Journal of Clinical Nutrition* **2004**, *79*, 424-429.
59. Nguyen, P.; Grajeda, R.; Melgar, P.; Marcinkevage, J.; Flores, R.; Martorell, R. Weekly may be as efficacious as daily folic acid supplementation in improving folate status and lowering serum homocysteine concentrations in guatemalan women. *Journal of Nutrition* **2008**, *138*, 1491-1498.
60. Nguyen, P.; Tam, C.; O'Connor, D.L.; Kapur, B.; Koren, G. Steady state folate concentrations achieved with 5 compared with 1.1 mg folic acid supplementation among women of childbearing age. *American Journal of Clinical Nutrition* **2009**, *89*, 844-852.
61. Briars, B.; Skeaff, M.; Adank, C.; Green, T. Once-a-week versus daily folic acid supplementation: Effects on red blood cell folate concentrations in women of child-bearing age [abstract]. In *New Zealand medical journal*, 2001; Vol. 114, p 454.
62. Olthof, M.R.; Bots, M.L.; Katan, M.B.; Verhoef, P. Effect of folic acid and betaine supplementation on flow-mediated dilation: A randomized, controlled study in healthy volunteers. *PLoS Clinical Trials* **2006**, *1*, e10.

63. Oort, F.V.; Melse-Boonstra, A.; Brouwer, I.A.; Clarke, R.; West, C.E.; Katan, M.B.; Verhoef, P. Folic acid and reduction of plasma homocysteine concentrations in older adults: A dose-response study. In *American journal of clinical nutrition*, 2003; Vol. 77, pp 1318-1323.
64. Papandreou, D.; Malindretos, P.; Arvanitidou, M.; Makedou, A.; Rousso, I. Oral supplementation of folic acid for two months reduces total serum homocysteine levels in hyperhomocysteinemic greek children. *Hippokratia* **2010**, *14*, 105-108.
65. Pathansali, R.; Mangoni, A.A.; Creagh-Brown, B.; Lan, Z.C.; Ngow, G.L.; Yuan, X.F.; Ouldred, E.L.; Sherwood, R.A.; Swift, C.G.; Jackson, S.H. Effects of folic acid supplementation on psychomotor performance and hemorheology in healthy elderly subjects. *Archives of Gerontology & Geriatrics* **2006**, *43*, 127-137.
66. Peña, A.S.; Wiltshire, E.; Gent, R.; Piotto, L.; Hirte, C.; Couper, J. Folic acid does not improve endothelial function in obese children and adolescents. In *Diabetes care*, 2007; Vol. 30, pp 2122-2127.
67. Petrogianni, M.; Grammatikaki, E.; Kalogeropoulos, N.; Peristeraki, A.; Moschonis, G.; Pitsavos, C.; Antonopoulou, S.; Manios, Y. Additional benefit in cvd risk indices derived from the consumption of fortified milk when combined with a lifestyle intervention. *Public Health Nutrition* **2014**, *17*, 440-449.
68. Pinto, X.; Vilaseca, M.A.; Balcells, S.; Artuch, R.; Corbella, E.; Meco, J.F.; Vila, R.; Pujol, R.; Grinberg, D. A folate-rich diet is as effective as folic acid from supplements in decreasing plasma homocysteine concentrations. *International Journal of Medical Sciences* **2005**, *2*, 58-63.
69. Pullin, C.H.; Ashfield-Watt, P.A.; Burr, M.L.; Clark, Z.E.; Lewis, M.J.; Moat, S.J.; Newcombe, R.G.; Powers, H.J.; Whiting, J.M.; McDowell, I.F. Optimization of dietary folate or low-dose folic acid supplements lower homocysteine but do not enhance endothelial function in healthy adults, irrespective of the methylenetetrahydrofolate reductase (c677t) genotype. *Journal of the American College of Cardiology* **2001**, *38*, 1799-1805.
70. Qin, X.; Li, J.; Cui, Y.; Liu, Z.; Zhao, Z.; Ge, J.; Guan, D.; Hu, J.; Wang, Y.; Zhang, F.*, et al.* Effect of folic acid intervention on the change of serum folate level in hypertensive chinese adults: Do methylenetetrahydrofolate reductase and methionine synthase gene polymorphisms affect therapeutic responses? *Pharmacogenetics and Genomics* **2012**, *22*, 421-428.
71. Quinlivan, E.P.; McPartlin, J.; McNulty, H.; Ward, M.; Strain, J.J.; Weir, D.G.; Scott, J.M. Importance of both folic acid and vitamin b12 in reduction of risk of vascular disease. *Lancet* **2002**, *359*, 227-228.
72. Raigani, M.; Yaghmaei, B.; Amirjannti, N.; Lakpour, N.; Akhondi, M.M.; Zeraati, H.; Hajihosseinal, M.; Sadeghi, M.R. The micronutrient supplements, zinc sulphate and folic acid, did not ameliorate sperm functional parameters in oligoasthenoteratozoospermic men. *Andrologia* **2014**, *46*, 956-962.
73. Rhode, B.M.; Cooper, B.A.; Farmer, F.A. Effect of orange juice, folic acid, and oral contraceptives on serum folate in women taking a folate-restricted diet. *Journal of the American College of Nutrition* **1983**, *2*, 221-230.
74. Rosenthal, J.; Milla, G.; Flores, A.; Yon, M.; Pfeiffer, C.; Umana, E.; Skerrette, N.; Barahona, F.; Cooperative Folic Acid Research, G. Effect of different dosage and administration schedules of folic acid on blood folate levels in a population of honduran women of reproductive age. *Public Health Nutrition* **2008**, *11*, 822-830.
75. Schorah, C.J.; Devitt, H.; Lucock, M.; Dowell, A.C. The responsiveness of plasma homocysteine to small increases in dietary folic acid: A primary care study. *European Journal of Clinical Nutrition* **1998**, *52*, 407-411.
76. Sharifi, F.; Fakhrzadeh, H.; Mirarefin, M.; Pourebrahim, R.; Nouri, M.; Forouzanfar, M.H.; Ghotbi, S.; Ghaderpanahi, M.; Larijani, B. The effects of high-dose folic acid on blood pressure of hypertensive adults with hyperhomocysteinemia: A randomized double- blind placebo controlled clinical trial (tehran homocysteine survey). *Iranian Journal of Diabetes and Lipid Disorders* **2010**, *9*, 13-20.
77. Sheu, W.H.; Chin, H.M.; Lee, W.J.; Wan, C.J.; Su, H.Y.; Lang, H.F. Prospective evaluation of folic acid supplementation on plasma homocysteine concentrations during weight reduction: A randomized, double-blinded, placebo-controlled study in obese women. *Life Sciences* **2005**, *76*, 2137-2145.

78. Shidfar, F.; Homayounfar, R.; Fereshtehnejad, S.M.; Kalani, A. Effect of folate supplementation on serum homocysteine and plasma total antioxidant capacity in hypercholesterolemic adults under lovastatin treatment: A double-blind randomized controlled clinical trial. *Archives of Medical Research* **2009**, *40*, 380-386.
79. Smidt, L.J.; Cremin, F.M.; Grivetti, L.E.; Clifford, A.J. Influence of folate status and polyphenol intake on thiamin status of irish women. *American Journal of Clinical Nutrition* **1990**, *52*, 1077-1082.
80. Strandhagen, E.; Landaas, S.; Thelle, D.S. Folic acid supplement decreases the homocysteine increasing effect of filtered coffee. A randomised placebo-controlled study. *European Journal of Clinical Nutrition* **2003**, *57*, 1411-1417.
81. Sweeney, M.R.; McPartlin, J.; Scott, J. Folic acid fortification and public health: Report on threshold doses above which unmetabolised folic acid appear in serum. *BMC Public Health* **2007**, *7*, 41.
82. Tapola, N.S.; Karvonen, H.M.; Niskanen, L.K.; Sarkkinen, E.S. Mineral water fortified with folic acid, vitamins b6, b12, d and calcium improves folate status and decreases plasma homocysteine concentration in men and women. *European Journal of Clinical Nutrition* **2004**, *58*, 376-385.
83. Tighe, P.; Ward, M.; McNulty, H.; Finnegan, O.; Dunne, A.; Strain, J.; Molloy, A.M.; Duffy, M.; Pentieva, K.; Scott, J.M. A dose-finding trial of the effect of long-term folic acid intervention: Implications for food fortification policy. *American Journal of Clinical Nutrition* **2011**, *93*, 11-18.
84. Title, L.M.; Cummings, P.M.; Giddens, K.; Genest, J.J.; Nassar, B.A.; Rocha, E. Effect of folic acid and antioxidant vitamins on endothelial dysfunction in patients with coronary artery disease. [portuguese]. *Revista Portuguesa de Cardiologia* **2000**, *19*, 1319-1320.
85. Toprak, A.; Erenus, M.; Ilhan, A.H.; Haklar, G.; Fak, A.S.; Oktay, A. The effect of postmenopausal hormone therapy with or without folic acid supplementation on serum homocysteine level. *Climacteric* **2005**, *8*, 279-286.
86. Tsui, J.C.; Nordstrom, J.W. Folate status of adolescents: Effects of folic acid supplementation. *Journal of the American Dietetic Association* **1990**, *90*, 1551-1556.
87. Tucker, K.L.; Olson, B.; Bakun, P.; Dallal, G.E.; Selhub, J.; Rosenberg, I.H. Breakfast cereal fortified with folic acid, vitamin b-6, and vitamin b-12 increases vitamin concentrations and reduces homocysteine concentrations: A randomized trial. *American Journal of Clinical Nutrition* **2004**, *79*, 805-811.
88. Ubbink, J.B.; Vermaak, W.H.; van der Merwe, A.; Becker, P.J.; Delport, R.; Potgieter, H.C. Vitamin requirements for the treatment of hyperhomocysteinem. *The Journal of nutrition* **1994**, *124*, 1927.
89. Ubbink, J.B.; van der Merwe, A.; Vermaak, W.J.; Delport, R. Hyperhomocysteinemia and the response to vitamin supplementation. *Clinical Investigator* **1993**, *71*, 993-998.
90. Ubbink, J.B.; Vermaak, W.J.; van der Merwe, A.; Becker, P.J. Vitamin b-12, vitamin b-6, and folate nutritional status in men with hyperhomocysteinemia. *American Journal of Clinical Nutrition* **1993**, *57*, 47-53.
91. Ubbink, J.B.; Vermaak, W.J.; Delport, R.; van der Merwe, A.; Becker, P.J.; Potgieter, H. Effective homocysteine metabolism may protect south african blacks against coronary heart disease. *American Journal of Clinical Nutrition* **1995**, *62*, 802-808.
92. Undas, A.; Domagala, T.B.; Jankowski, M.; Szczeklik, A. Treatment of hyperhomocysteinemia with folic acid and vitamins b12 and b6 attenuates thrombin generation. *Thrombosis Research* **1999**, *95*, 281-288.
93. Venn, B.J.; Green, T.J.; Moser, R.; McKenzie, J.E.; Skeaff, C.M.; Mann, J. Increases in blood folate indices are similar in women of childbearing age supplemented with [6s]-5-methyltetrahydrofolate and folic acid. *Journal of Nutrition* **2002**, *132*, 3353-3355.
94. Venn, B.J.; Mann, J.I.; Williams, S.M.; Riddell, L.J.; Chisholm, A.; Harper, M.J.; Aitken, W.; Rossaak, J.I. Assessment of three levels of folic acid on serum folate and plasma homocysteine: A randomised placebo-controlled double-blind dietary intervention trial. *European Journal of Clinical Nutrition* **2002**, *56*, 748-754.
95. Venn, B.J.; Green, T.J.; Moser, R.; Mann, J.I. Comparison of the effect of low-dose supplementation with l-5-methyltetrahydrofolate or folic acid on plasma homocysteine: A randomized placebo-controlled study. *American Journal of Clinical Nutrition* **2003**, *77*, 658-662.

96. Vermeulen, E.G.; Stehouwer, C.D.; Twisk, J.W.; van den Berg, M.; de Jong, S.C.; Mackaay, A.J.; van Campen, C.M.; Visser, F.C.; Jakobs, C.A.; Bulterjis, E.J.*, et al.* Effect of homocysteine-lowering treatment with folic acid plus vitamin b6 on progression of subclinical atherosclerosis: A randomised, placebo-controlled trial. *Lancet* **2000**, *355*, 517-522.
97. van Vliet, T.; Jacobs, R.G.; de Deckere, E.; van den Berg, H.; de Bree, A.; van der Put, N.M. Effect of fortified spread on homocysteine concentration in apparently healthy volunteers. *European Journal of Clinical Nutrition* **2007**, *61*, 769-778.
98. von der Porten, A.E.; Gregory, J.F., 3rd; Toth, J.P.; Cerda, J.J.; Curry, S.H.; Bailey, L.B. In vivo folate kinetics during chronic supplementation of human subjects with deuterium-labeled folic acid. *Journal of Nutrition* **1992**, *122*, 1293-1299.
99. Voutilainen, S.; Rissanen, T.H.; Virtanen, J.K.; Porkkala-Sarataho, E.; Kaikkonen, J.; Seppanen, K.; Tuomainen, T.P.; Lehtimaki, T.; Rontu, R.; Hamelahti, P.*, et al.* Folic acid supplementation increases serum paraoxonase activity: Evidence from a randomized double blind oral supplementation trial in men. In *Current topics in nutraceutical research*, 2003; Vol. 1, p 175.
100. Wald, D.S.; Bishop, L.; Wald, N.J.; Law, M.; Hennessy, E.; Weir, D.; McPartlin, J.; Scott, J. Randomized trial of folic acid supplementation and serum homocysteine levels. *Archives of Internal Medicine* **2001**, *161*, 695-700.
101. Wang, L.; Li, H.; Zhou, Y.; Jin, L.; Liu, J. Low-dose b vitamins supplementation ameliorates cardiovascular risk: A double-blind randomized controlled trial in healthy chinese elderly. *European Journal of Nutrition* **2015**, *54*, 455-464 410p.
102. Ward, M.; McNulty, H.; McPartlin, J.; Strain, J.J.; Weir, D.G.; Scott, J.M. Plasma homocysteine, a risk factor for cardiovascular disease, is lowered by physiological doses of folic acid. *Qjm* **1997**, *90*, 519-524.
103. Williams, E.; Stewart-Knox, B.; Bradbury, I.; Rowland, I.; Pentieva, K.; Helander, A.; McNulty, H. Effect of folic acid supplementation on mood and serotonin response in healthy males. *British Journal of Nutrition* **2005**, *94*, 602-608.
104. Winkels, R.M.; Brouwer, I.A.; Clarke, R.; Katan, M.B.; Verhoef, P. Bread cofortified with folic acid and vitamin b-12 improves the folate and vitamin b-12 status of healthy older people: A randomized controlled trial. *American Journal of Clinical Nutrition* **2008**, *88*, 348-355.
105. Wolters, M.; Hermann, S.; Hahn, A. Effect of multivitamin supplementation on the homocysteine and methylmalonic acid blood concentrations in women over the age of 60 years. *European Journal of Nutrition* **2005**, *44*, 183-192.
106. Woo, K.S.; Chook, P.; Chan, L.L.; Cheung, A.S.; Fung, W.H.; Qiao, M.; Lolin, Y.I.; Thomas, G.N.; Sanderson, J.E.; Metreweli, C.*, et al.* Long-term improvement in homocysteine levels and arterial endothelial function after 1-year folic acid supplementation. *American Journal of Medicine* **2002**, *112*, 535-539.
107. Wright, A.J.; King, M.J.; Wolfe, C.A.; Powers, H.J.; Finglas, P.M. Comparison of (6 s)-5-methyltetrahydrofolic acid v. Folic acid as the reference folate in longer-term human dietary intervention studies assessing the relative bioavailability of natural food folates: Comparative changes in folate status following a 16-week placebo-controlled study in healthy adults. *British Journal of Nutrition* **2010**, *103*, 724-729.
108. Zheng, S.F.; Ershow, A.G.; Yang, C.S.; Li, G.Y.; Li, R.S.; Li, H.; Zou, X.L.; Liu, X.F.; Song, L.H.; Qing, Q.S.*, et al.* Nutritional status in linxian, china: Effects of season and supplementation. *International Journal for Vitamin & Nutrition Research* **1989**, *59*, 190-199.
109. Higgins JPT; Altman DG; Sterne JAC. Chapter 8: Assessing risk for bias in included studies. In *Cochrane handbook for systematic reviews of interventions. 5.1.0*, Higgins, J., Green, S, Ed. The Cochrane Collaboration: March 2011.
110. Viswanathan M; Berkman ND. Development of the rti item bank on risk of bias and precision of observational studies. *Journal of clinical epidemiology* **2012**, *65*, 163-178.

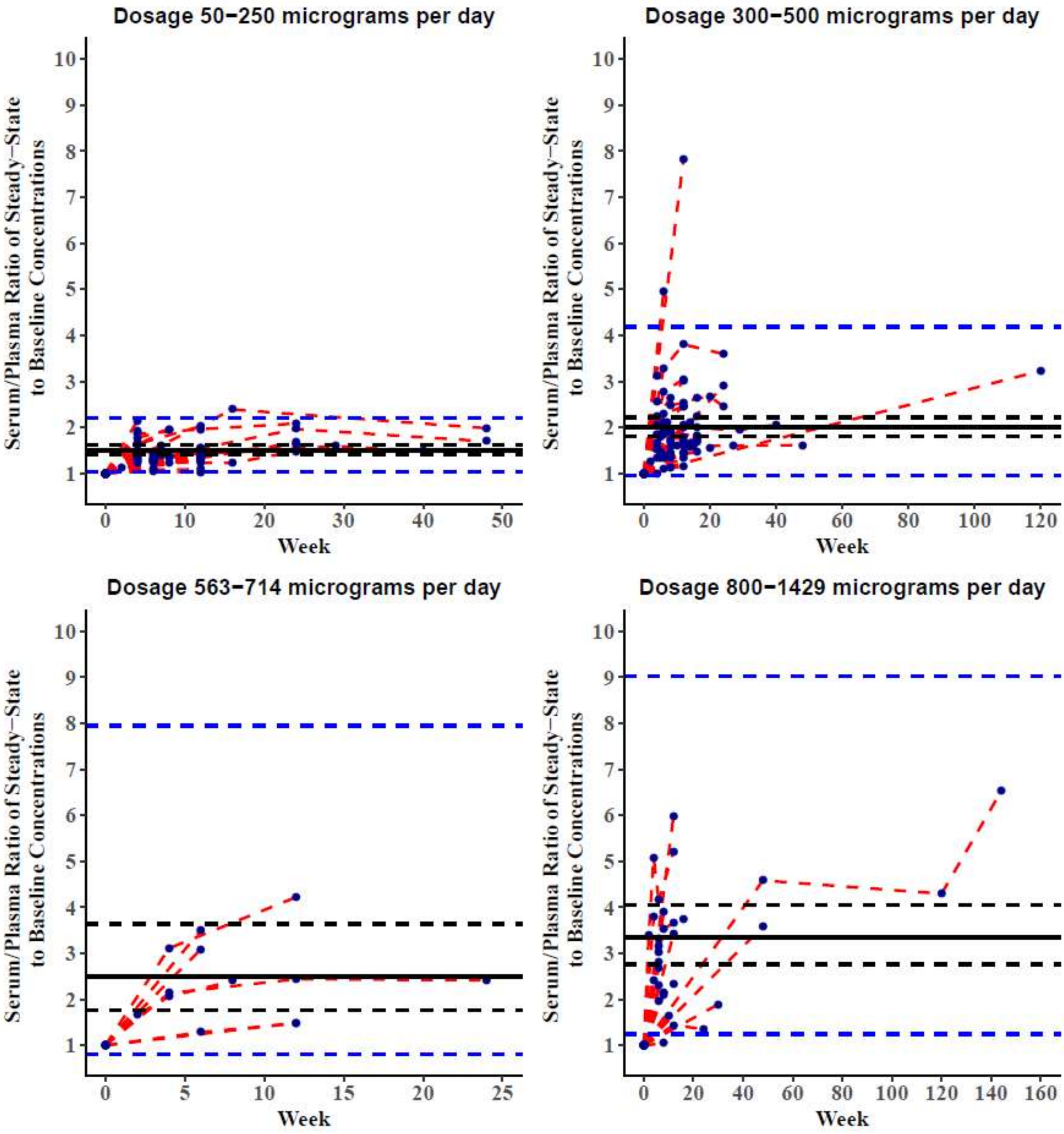

Figure S1: Fold change in serum/plasma folate concentrations from baseline over time in weeks for each study extracted. Blue dots: each data point. Black line: the estimated fold change at steady-state. Black dashed lines: 95% crediable intervals. Blue dashed lines: 95% posterior predictive intervals.

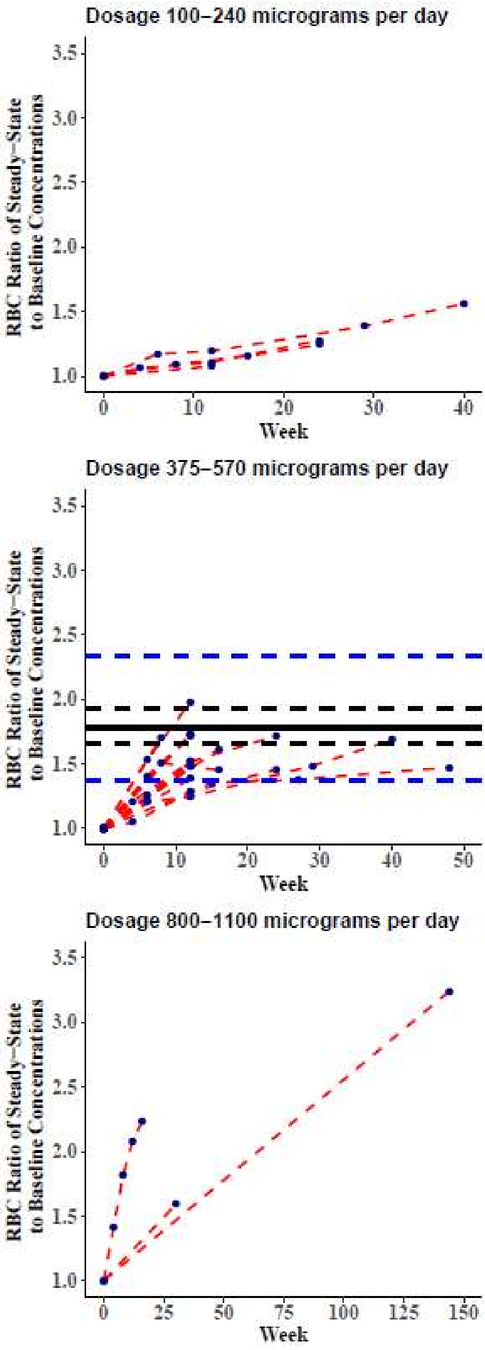

Figure S2: Fold change in red blood cell (RBC) folate concentrations from baseline over time in weeks for each study extracted. Blue dots: each data point. Black line: the estimated fold change at steady-state. Black dashed lines: 95% crediable intervals. Blue dashed lines: 95% posterior predictive intervals.
